# Surface wave elastography is a reliable method to correlate muscle elasticity, torque, and electromyography activity level

**DOI:** 10.14814/phy2.14955

**Published:** 2021-08-02

**Authors:** Gustavo A. Grinspan, Hélio V. Cabral, Leonardo M. L. de Souza, Liliam F. de Oliveira, Sofía Aguiar, Ernesto Blanco, Nicolás Benech

**Affiliations:** ^1^ Sección Biofísica y Biología de Sistemas Facultad de Ciencias Universidad de la República Montevideo Uruguay; ^2^ Laboratorio de Acústica Ultrasonora Facultad de Ciencias Universidad de la República Montevideo Uruguay; ^3^ Centre of Precision Rehabilitation for Spinal Pain (CPR Spine) School of Sport, Exercise and Rehabilitation Sciences College of Life and Environmental Sciences University of Birmingham Birmingham UK; ^4^ Programa de Engenharia Biomédica (COPPE) Universidade Federal do Rio de Janeiro Rio de Janeiro Brazil; ^5^ Instituto de Ensayo de Materiales Facultad de Ingeniería Universidad de la República Montevideo Uruguay; ^6^ Instituto de Física Facultad de Ciencias Universidad de la República Montevideo Uruguay

**Keywords:** elbow flexion torque, EMG activity, non‐ultrasound surface wave elastography (NU‐SWE), shear elastic modulus (*c*_55_), skeletal muscle

## Abstract

The shear elastic modulus is one of the most important parameters to characterize the mechanical behavior of soft tissues. In biomechanics, ultrasound elastography is the gold standard for measuring and mapping it locally in skeletal muscle in vivo. However, their applications are limited to the laboratory or clinic. Thus, low‐frequency elastography methods have recently emerged as a novel alternative to ultrasound elastography. Avoiding the use of high frequencies, these methods allow obtaining a mean value of bulk shear elasticity. However, they are frequently susceptible to diffraction, guided waves, and near field effects, which introduces biases in the estimates. The goal of this work is to test the performance of the non‐ultrasound surface wave elastography (NU‐SWE), which is portable and is based on new algorithms designed to correct the incidence of such effects. Thus, we show its first application to muscle biomechanics. We performed two experiments to assess the relationships of muscle shear elasticity versus joint torque (*experiment 1*) and the electromyographic activity level (*experiment 2*). Our results were comparable regarding previous works using the reference ultrasonic methods. Thus, the NU‐SWE showed its potentiality to get wide the biomechanical applications of elastography in many areas of health and sports sciences.

## Graphical abstract

This work describes the non‐ultrasound surface wave elastography (NU‐SWE) as a novel tool for muscle biomechanics research. The method is low‐cost, portable, and can provide an accurate estimate of the bulk elasticity of skeletal muscle in real‐time. To test the performance of the NU‐SWE, we performed two experiments to assess the relationship between the muscle shear elasticity versus joint torque (*experiment 1*) and the electromyography activity level (*experiment 2*).

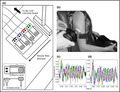

**New and Noteworthy**

This work describes the non‐ultrasound surface wave elastography as a novel tool for muscle biomechanics research. It is a low‐frequency method whose main innovation lies in its inversion algorithm. This corrects the incidence of the near field effects and guided wave propagation, thus obtaining a muscle shear elastic modulus comparable to ultrasound elastography. Besides, it is a low‐cost, real‐time, and portable method, making it ideal to extend the current biomechanical applications of elastography.

## 1 INTRODUCTION

Assessing the shear elasticity of skeletal muscle in vivo and in a reliable way is of great interest within different areas of life and health sciences. This parameter provides valuable information about the different processes and biological phenomena associated with the intrinsic mechanical state of the tissue (Sarvazyan, 1993). Thus, in recent years, the elastography field has improved the technical capability of biomechanical studies, providing useful methods to evaluate the mechanical properties of skeletal muscle in vivo.

Particularly, ultrasonic methods such as transient elastography (TE; Catheline et al., 1999) and supersonic shear imaging (SSI; Bercoff et al., 2004), have been positioned as the reference methods in biomechanics to characterize muscle shear elasticity. Indeed, there are several recent works where these methods were applied to address complex problems in muscle biomechanics, such as the load sharing between muscles, muscular fatigue, and the relation of muscle shear elasticity with joint torque and electromyography (EMG) activity level (Ateş et al., 2015; Bouillard, Hug, et al., 2012; Bouillard, Nordez, et al., 2012; Bouillard, Nordez, & Hug, 2011; Gennisson et al., 2005, 2010; Lapole et al., 2015; Nordez et al., 2009; Nordez & Hug, 2010; Yoshitake et al., 2014). These studies were performed applying ultrasound elastography and other traditional methods used in the muscle biomechanics field (e.g., isokinetic dynamometry, surface EMG). The main advantages of TE and SSI methods are that they combine high‐frequency ultrasonic waves (10^6^ Hz), which exhibit a good spatial resolution (<1 mm), with low‐frequency waves (100~1000 Hz) that exhibit a good contrast in the shear elastic modulus. Nevertheless, they need some infrastructure (e.g., a clinic or laboratory) to be used properly. So, elastography into biomechanical research is currently limited to specific applications performed in clinics or laboratories. Besides, the relatively high cost of this technology restricts its availability, especially in laboratories and clinics with lower resources. Therefore, a current need in biomechanics is to develop new elastography methods that can provide solutions to these issues.

One alternative is the exclusive use of low‐frequency waves (~100 Hz). When the ultrasound frequencies are removed, the spatial information is lost. However, the information about elasticity remains. This allows reporting a numerical value about the bulk elasticity of the tissue. In this way, recent works refer to new elastography methods that use surface waves (or Rayleigh waves) to assess the mechanical properties of skeletal muscles (Benech et al., 2012; Courage, 2003; Grinspan et al., 2016; Sabra et al., 2007; Salman & Sabra, 2013). The general idea of these methods is to use a linear array of vibration sensors aligned with an external wave source, to record the surface displacement of the Rayleigh wave. Its velocity has a simple relation with the shear elasticity of the tissue. However, this relationship only holds in a semi‐infinite medium and the far‐field of the source (Benech et al., 2019). Since real muscles are finite, and the low‐frequency waves attenuate rapidly in soft tissues (Zhang, 2016), the wave propagation is often measured in the near‐field of the source. Thus, near‐field effects must be considered when computing the velocity. These effects include complex solutions to the Rayleigh secular equation. The complex solutions give rise to a propagating surface wave whose amplitude decays exponentially, termed as the leaky surface wave (Benech et al., 2017). Here, the Rayleigh wave interferes with the leaky wave, having consequences on the phase velocity of the surface field (Benech et al., 2017, 2019). Thus, the exclusive use of low frequencies introduces biases into the estimation of muscle shear elastic modulus. This is due to the incidence of the near field effects and guided wave propagation.

In this context, this work shows a new low‐frequency elastography method and its application to muscle biomechanics. This comprises substantial innovations compared with previous versions (Benech et al., 2012; Grinspan et al., 2016), to obtain reliable estimations of muscle shear elasticity. To emphasize that no ultrasound frequencies are involved, we refer to it as non‐ultrasound surface wave elastography (NU‐SWE). The method is low‐cost and allows measuring the skeletal muscle shear elasticity in vivo, in real‐time and in a noninvasive way. Besides, it is portable and can be used outside of a laboratory or clinic. The main innovation of such a method lies in the inversion algorithm to calculate the shear wave velocity from the surface displacement field. This algorithm considers the interference between the leaky and Rayleigh waves in the near field of the source and their incidence on the phase velocity (Benech et al., 2017, 2019). This issue is being the object of new developments in the field of elastography (Pitre et al., 2019). Thus, the NU‐SWE can relate the velocity of surface waves to the shear elastic modulus in a transversely isotropic medium like skeletal muscle. Then, the estimation of the shear elasticity is not biased by the guided wave and near field effects.

This work is the first to assess the reliability of the NU‐SWE for biomechanical research. In this sense, the main goal is to test the performance of the method, to provide reliable values of muscle shear elasticity under similar conditions to those of previous biomechanical studies using ultrasound elastography. For such purpose, we carried out two experiments to evaluate the relationship between the shear elasticity of skeletal muscle versus joint torque (*experiment 1*) and the EMG activity level (*experiment 2*). Thus, we expect that the viability of the innovative NU‐SWE method could extend elastography research applications in muscle biomechanics and related fields, mainly where the applicability of existing methods is not feasible or possible.

## 2 MATERIALS AND METHODS

### 2.1 Subjects

Eighteen healthy volunteers participated in *experiment 1* (nine men, nine women, age 26.78 ± 4.56 years, height 170.9 ± 9.69 cm, weight 66.75 ± 12.34 kg). Six healthy volunteers participated in *experiment 2* (four men, two women, age 27.3 ± 3.2 years, height 168.2 ± 9.9 cm, weight 69.4 ± 14.5 kg). The experimental design of the study was performed according to the last version of the Helsinki statement (2004) and approved by the Ethics Committees of the Faculty of Medicine (UdelaR, Uruguay) and the Clementino Fraga Filho University Hospital (HUCFF/UFRJ, Brazil).

### 2.2 Instrumentation

#### 2.2.1 Ergometry

**FIGURE 1:**
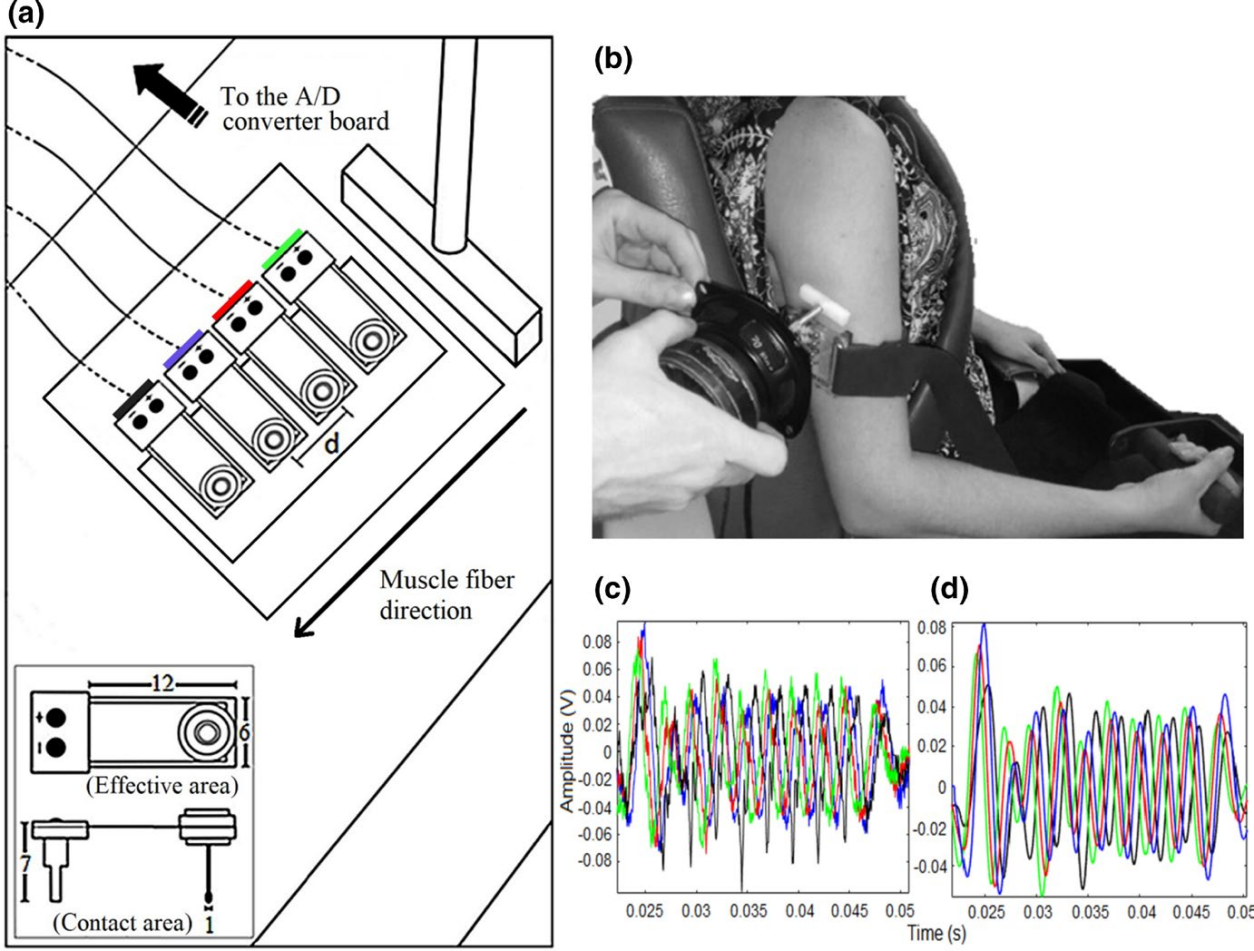
(a) Scheme showing the relative arrangement of the wave source, the underlying muscle fibers direction, and the linear array of vibration sensors (dimensions in mm). (b) An example of the non‐ultrasound surface wave elastography prototype placed over the free surface of the TB muscle. (c, d) Raw and filtered signals recorded by their corresponding sensor on the linear array (indicated by colors), respectively

**FIGURE 2:**
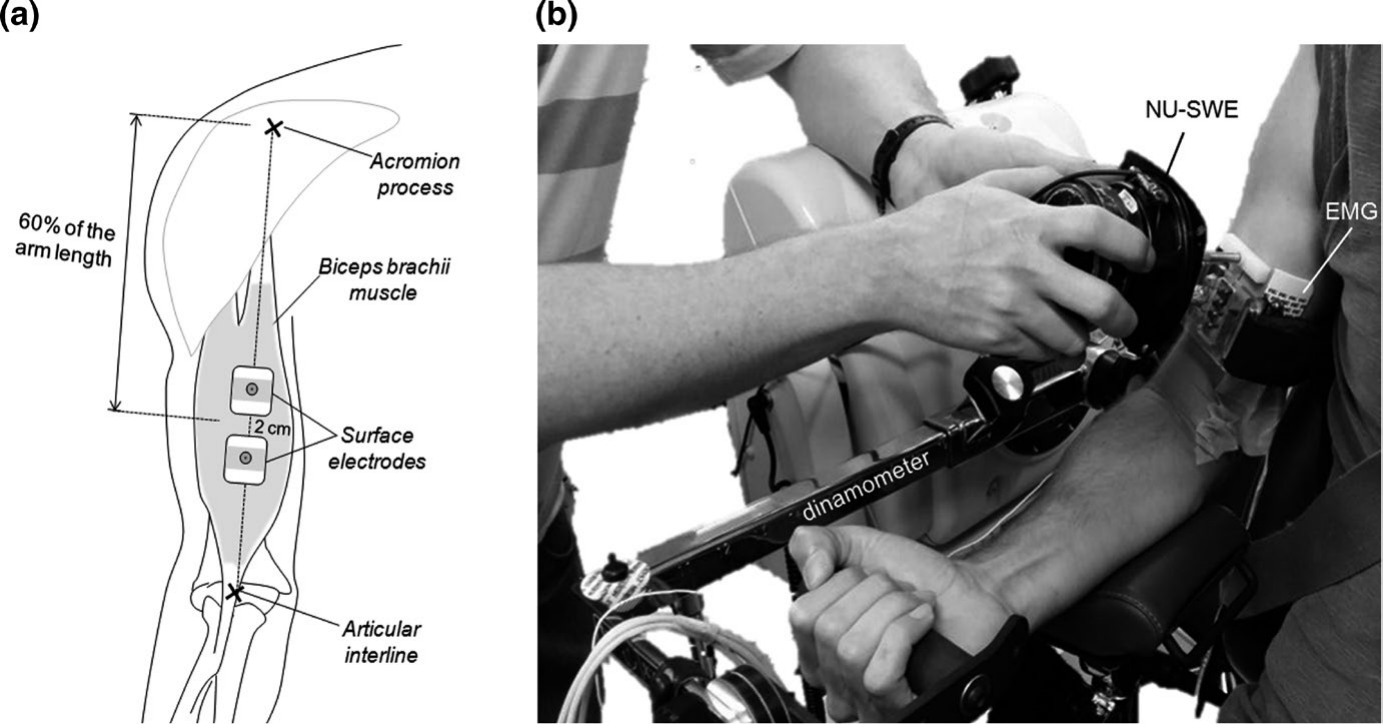
(a) Scheme of the electromyography (EMG) electrodes positioning over the biceps brachii muscle in *experiment 2*. (b): volunteer sitting on the isokinetic dynamometer to perform *experiments 1* and *2*. The relative placement of the wave source, the EMG bipolar electrodes, and the linear array of vibration sensors employed in *experiment 2* are shown

A research isokinetic dynamometer (Biodex System 4; Biodex) was used to measure the angle and torque of the elbow joint in *experiments 1* and *2*. During all data collection, the subjects were comfortably seated on the dynamometer with their elbow coaxially aligned with the dynamometer axis of rotation and flexed at 90º, and their wrist in a supine position (Figures 1b and 2b).

#### 2.2.2 Non‐ultrasound surface wave elastography

The NU‐SWE method consists of exciting the propagation of low‐amplitude and low‐frequency surface waves (~100 Hz) at the free surface of the muscle, recording their displacement and estimating their phase velocity. Then, the method retrieves the shear wave velocity, which is related to the muscle shear elastic modulus.

In this work, a homemade NU‐SWE prototype was used to perform *Experiment 1* on biceps brachii (BB) and triceps brachii lateral head (TB) muscles, as well as *Experiment 2* on BB muscle. The NU‐SWE device is composed of a linear array of contact vibration sensors, an external wave source, an audio amplifier, an analog‐to‐digital (A/D) converter board (NI‐USB 6009; National Instruments), and a computer. In this case, the external wave source, a shaker with a coupled piston, was driven by ten cycles of a sinusoid with a central frequency varying between 50 and 250 Hz. It vibrates normally to the free surface of the muscle to excite mainly the vertical component of surface waves (Figure 1b). This component of the vibrations is recorded by the linear array of sensors, each constituted by a piezoelectric PVDF flexible film. Besides, they possess a mass on the end with a small extension attached to it (Figure 1a). In this way, the NU‐SWE uses point‐like sensors (contact area ~1 mm^2^), thus avoiding diffraction effects at the reception (Benech et al., 2012; Grinspan et al., 2016). Furthermore, as is shown in Figure 1a, the source is aligned with the array of sensors, which must be equally spaced a distance d from each other. According to this, the sensors are arranged on a plate that holds them in the correct configuration and positioned on the arm by a holding brace (Figures 1a and 1b). Thus, the vibration is captured sequentially by the sensors, producing a signal that is digitized by the A/D converter board and transferred to a computer for processing.

Unlike other low‐frequency elastography methods, the data processing of the NU‐SWE involves the use of inversion algorithms designed to automatically correct the incidence of the guided wave propagation and near field effects. In this way, the method estimates the shear elasticity of the muscle by computing a reliable value of the shear wave velocity (*V*_T_) in a linear regime. The inversion algorithms, as well as the signal processing algorithm, are detailed in Appendix A.

If we consider the muscle like a transversely isotropic solid (Gennisson et al., 2005; Nordez & Hug, 2010), the longitudinal shear elastic modulus (*c*_55_) is related to a shear wave propagating in the muscle fibers direction with perpendicular polarization $V_{T}^{\|}$ = $\sqrt{c_{55}/\rho }$ (where ρ is the muscle mass density = 1000 kg/m^3^; Benech et al., 2019). Due to the arrangement of the wave source and the linear array of vibration sensors (Figure 1a), the NU‐SWE retrieves $V_{T}^{\|}$. Thus, in an analogous way to previous works using ultrasound elastography (Ateş et al., 2015; Bouillard, Hug, et al., 2012; Bouillard, Nordez, et al., 2012; Bouillard et al., ,^2011^; Gennisson et al., 2005, 2010; Lapole et al., 2015; Nordez et al., 2009; Nordez & Hug, 2010; Yoshitake et al., 2014), we can estimate the muscle shear elasticity as follows:

(1) $$c_{55}=\rho V_{T}^{\|2}.$$

This equation assumes that the viscous effects of the tissue are negligible. So, the NU‐SWE, as classic elastography studies using echographic and magnetic resonance imaging, assumes that the mechanical behavior of muscle is like that of a linear elastic material (Ateş et al., 2015; Bercoff et al., 2004; Catheline et al., 2004; Deffieux et al., 2009; Gennisson et al., 2003, 2005; Heers et al., 2003; Jenkyn et al., 2003; Nordez et al., 2008; Nordez & Hug, 2010; Tanter et al., 2008; Uffmann et al., 2004).

#### 2.2.3 Surface EMG

In *Experiment 2*, bipolar EMGs were collected from the short head of BB muscle with a pair of electrodes (20 mm interelectrode distance; 2223BRQ; 3M Company). First, the acromion process and the articular interline of the elbow joint were identified and the distance between them was considered to define the arm length. The electrodes pair was then positioned 60% distally from the acromion and parallel to the BB fibers (Figure 2a). Since the bipolar EMGs amplitude is markedly small when detected nearby to the muscle innervation zone (IZ; Nishihara et al., 2013; Rainoldi et al., 2004), the IZ location of BB muscle was identified before the placement of electrodes. During this procedure, the EMGs were visually inspected with a dry array of sixteen silver bar electrodes (10 mm interelectrode distance; LISiN‐Politecnico di Torino) while the participants were asked to gently isometrically contract their elbow flexors. Specifically, the array was placed over the line connecting the acromion and the elbow articular interline, and its position was slightly changed until propagation of action potentials of individual motor units could be clearly observed across electrodes. The IZ region was then identified as the single‐differential channel with a small amplitude and from which it was possible to observe the action potentials propagation (cf. figure 2 in Rainoldi et al., 2004). If the IZ location corresponded with the predefined placement of electrodes (at 60% of the arm's length; Figure 2a), the bipolar electrodes were positioned proximally. The reference electrode was placed at the olecranon and, before positioning detection electrodes, the skin was shaved and cleaned with abrasive paste.

Surface EMGs were amplified with a variable gain (10–500 Hz bandwidth amplifier, common‐mode rejection ratio >100 dB; EMG‐USB2; OT Bioelettronica) and digitized at 2048 samples/s using a 12‐bit A/D converter with 5 V dynamic range.

### 2.3 Experimental protocol

#### 2.3.1 Muscle thickness and maximal voluntary contraction measurements

At the beginning of both *experiments*, the thickness of tested muscles was measured at 60% of the arm's length distally from the acromion. This was done using ultrasonic images acquired with a portable ultrasound scanner (Hitachi EUB‐405). Such measure was performed to obtain an accurate value of $V_{T}^{\|}$ through the inversion algorithms described in Appendix A. Additionally, also in both *experiments*, the volunteers performed two maximal isometric voluntary elbow flexions with the elbow flexed at 90° (each lasting 5 s and resting 90 s between them), to determine the maximal voluntary contraction (MVC). The highest MVC value was assumed as the maximal elbow flexion torque and was used to normalize submaximal contractions, as detailed subsequently.

#### Experiment 1

The subjects were seated on the dynamometer and the NU‐SWE was placed at the midline of the muscle belly, on the same arm location where the muscle thickness was collected (Figure 2b). Thus, at the beginning of the test, the *c*_55_ of the resting BB and TB muscles were measured individually using NU‐SWE. Next, the volunteers were asked to perform three isometric flexions at 10%, 20%, and 30% of MVC (each lasting 20 s and resting 90 s between them) in random order, using real‐time visual feedback of the torque signal. Parallel to the isometric flexions, the *c*_55_ of the BB and TB muscles was measured in random order by using the NU‐SWE. Since the current prototype does not allow measuring two or more muscles at the same time, such measurements were not performed simultaneously. Each 20 s task described above consisted of ten individual measurements (i.e., sample rate of 0.5 Hz) of *c*_55_ for each discrete torque level (0 [rest], 10%, 20%, and 30% of MVC). Likewise, to assess the repeatability of the method, the subjects were asked to perform two series of each task.

#### Experiment 2

In this experiment, isometric elbow flexion contractions with a real‐time continuous torque‐varying profile were performed by the volunteers. Specifically, they were asked to increase elbow flexion torque from rest to 40% MVC in 20 s, then to hold it at the reached level for 5 s, and return to rest in 20 s. A total of four trapezoidal profiles were considered with a 120‐s rest interval between them. Two series were performed for the acquisition of EMG activity versus torque profile in an alternated way with another two series for the comparison of *c*_55_ versus torque. Visual feedback of elbow flexion torque was presented on a computer's screen positioned 1 m ahead of the participants. Data collection started after participants had familiarized with visual feedback and could successfully follow the trapezoidal profiles.

### 2.4 Data analysis

For *experiment 1*, we assess the relationship between *c*_55_ and torque, both for BB and TB muscle of each subject. The ten signals obtained from the respective tasks were used to calculate an average value of *c*_55_ and their corresponding standard deviation. This was done for all volunteers in the two series. Thus, from these individual mean values, we obtained an overall elasticity of the population for both series of each task.

In *experiment 2*, bipolar EMGs were band‐pass filtered with a fourth‐order Butterworth filter (15–350 Hz cut‐off frequencies; zero lag, bidirectional filter). Then, the root mean square (RMS) was calculated over ~70 ms epochs, providing a total of 65 RMS values per trapezoidal profile. These RMS values were normalized with respect to the maximal RMS along the ramp. In order to obtain the same number of points over time for the NU‐SWE's estimates, the shear elasticity of the BB was also sampled at 1.4 Hz (65 values per ramp).

### 2.5 Statistical analysis

Linear regressions were performed between the data collected during tasks for all subjects (*c*_55_ vs. torque, for the first and second series of *experiment 1*; *c*_55_ vs. normalized EMG RMS, for both series of the respective profiles of *experiment 2*). Also, we performed a linear regression from the complete set of data of *experiment 2* (group analysis). The coefficients of determination (*R* ^2^) were calculated in each case to assess the goodness of the fit. Besides, for *experiment 2*, the regressions coefficients were used to estimate *c*_55_ at 0.3 and 0.7 of maximal EMG RMS activity.

Likewise, we assess the repeatability of *c*_55_ within each task of both experiments. In *experiment 1*, this was done from the values collected for each of the four torque levels. In *experiment 2*, we used the values estimated for the two EMG activity levels previously described. In this way, for both experiments, we calculated the intraclass correlation coefficient (ICC), the standard error measurement (SEM), and the coefficient of variation (CV) between the two series of each task (Hopkins, 2000).

To compare *c*_55_ between the two series of isometric contractions in *experiments 1* and *2*, we performed a repeated‐measures ANOVA (independent variable: *N°* of series [first, second]; dependent variable: *c*_55_) by using PAST 3.21 (Hammer et al., 2001). The same approach was used to compare the values of *c*_55_ according to the corresponding contraction intensity level (independent variable: torque, dependent variable: *c*_55_ (*experiment 1*); independent variable: EMG RMS activity level, dependent variable: *c*_55_ (*experiment 2*)). The critical level of significance was set at *p* < 0.05.

## 3 RESULTS

### 3.1 Experiment 1

The BB and TB muscles showed an opposite behavior concerning its *c*_55_—elbow flexion torque relationship. While the BB showed a linear increase in *c*_55_ with respect to elbow flexion torque (the average coefficient of determination was *R* ^2^ = 0.97 ± 0.02, both for the first and second series), the TB did not show significant changes. From 0% to 30% of MVC, the *c*_55_ of BB ranged from 4.57 to 109.24 kPa (first series) and 4.61 to 101.66 kPa (second series). In TB the variation was from 3.92 to 13.84 kPa (first series) and 4.15 to 15.97 kPa (second series). Table 1 summarizes the global results in the two series of each task for both muscles.

**TABLE 1:** Global results of *experiment 1*. Average values and SD (between brackets) of *c*_55_ of the biceps brachii (BB) and triceps brachii (TB) muscles obtained from both series of each task. The results of the repeatability analysis are also displayed

% MVC	BB				TB			
	0	10	20	30	0	10	20	30
								
$c_{55}$(kPa) First series	9.63 (3.61)	32.42 (7.43)	49.25 (9.18)	70.13 (15.89)	8.23 (2.26)	8.53 (2.82)	8.75 (3.19)	9.33 (3.44)
*c*_55_ (kPa) Second series	9.17 (3.35)	32.19 (7.23)	49.91 (8.70)	69.99 (13.60)	8.38 (2.62)	8.66 (3.04)	8.72 (3.05)	9.26 (3.66)
ICC	0.96	0.96	0.92	0.97	0.97	0.95	0.95	0.98
SEM	0.69	1.44	2.49	2.53	0.42	0.66	0.70	0.50
CV (%)	7.23	4.58	5.01	3.62	5.16	7.73	8.2	5.32

Abbreviation: CV, coefficient of variation.

The high ICC and low SEM values obtained denote the high reproducibility of the NU‐SWE to estimate *c*_55_ at different contraction intensity levels. Likewise, the CV between the two series was lower than 10%, both for the BB and TB. Regarding the comparison of *c*_55_ with respect to elbow flexion torque level, a significant main effect of contraction intensity was found for BB in the series 1 and 2 (*p* = 0.000, both cases). This indicates that *c*_55_ was substantially higher as the elbow flexion torque level increased. On the contrary, this main effect was not found for TB, where the differences in series 1 and 2 were not significant (*p* = 0.12 and 0.25, respectively). Additionally, the results of *c*_55_ measured at BB and TB were not significantly different between the two series of each task (*p* = 0.92 and 0.72, respectively). Tables S1 and S2 (https://figshare.com/s/998e925bcab5ed670d00) contain the complete set of data obtained in this experiment.

### 3.2 Experiment 2

**FIGURE 3:**
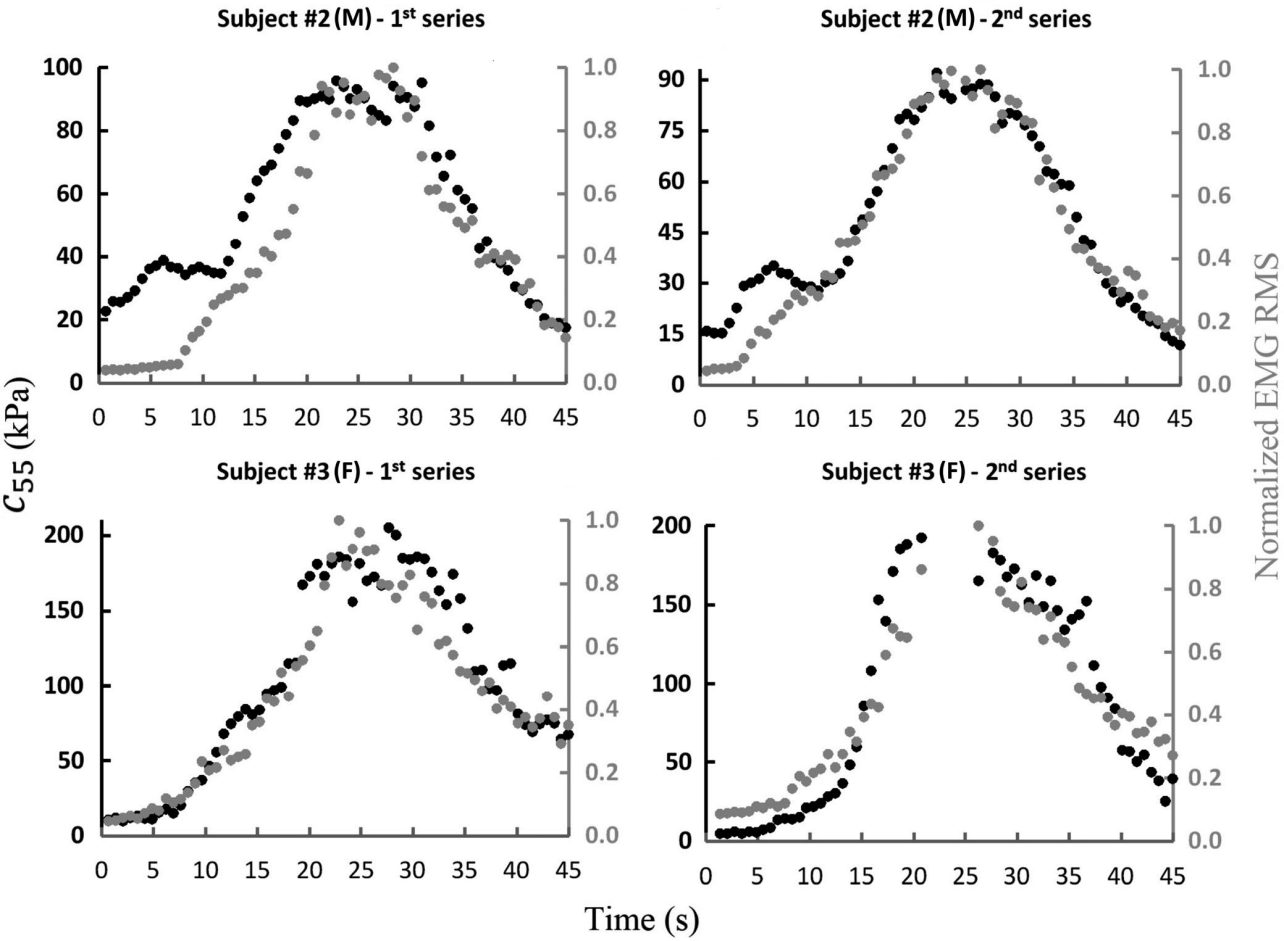
Two representative results of *c*_55_ and normalized electromyography root mean square of the biceps brachii recorded during both series of the isometric trapezoidal profiles. F, female; M, male.

**FIGURE 4:**
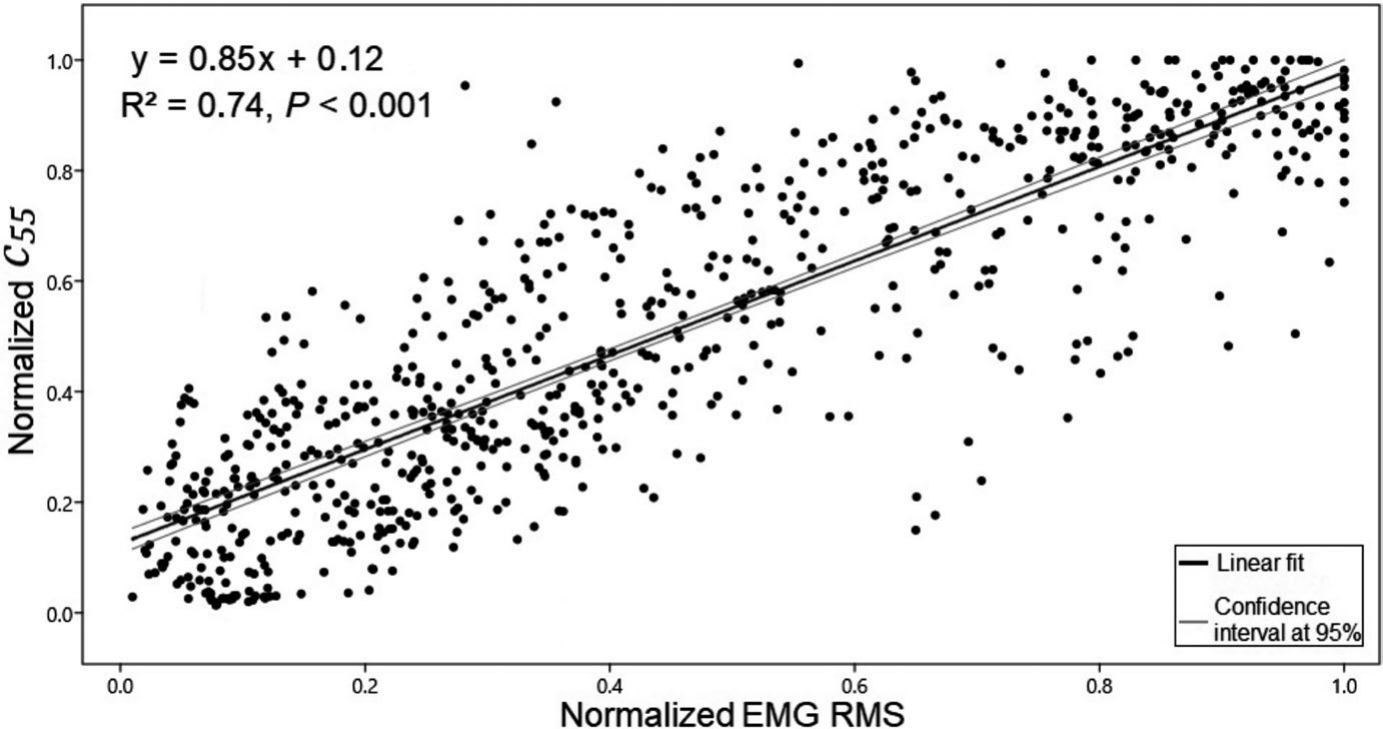
Group analysis performed over the complete set of normalized data collected from the biceps brachii muscle during the isometric trapezoidal profiles (*n* = 751). The *c*_55_ and the electromyography root mean square (EMG RMS) measured in all subjects show a significant linear relationship. Based on the level of confidence established, the regression coefficients of the linear fit are reliable

Although it is possible to observe some occasional, specific mismatches, a very good agreement was found between the trapezoidal profiles described by the NU‐SWE method and the EMGs (Figure 3). As is shown in Table 2, a significant linear relationship (*p* < 0.001) was verified between the EMG activity and *c*_55_ for all subjects in each series (*R* ^2^ = 0.82 ± 0.08, ranged from 0.71 and 0.93). When considering the complete set of data, this linear relationship was also found in the group analysis of Figure 4. The complete dataset of this experiment is available in Tables S3–S10 (https://figshare.com/s/998e925bcab5ed670d00).

**TABLE 2:** Determination and regression coefficients for each subject in both series of trapezoidal profiles. These coefficients were obtained from the linear fit performed between *c*_55_ (*y*) and EMG RMS normalized (*x*) values (*y* = *ax* + *b*)

No. of subjects	Sex	First series			Second series		
		*a*	*b*	*R* ^2*^	*a*	*b*	*R* ^2*^
1	M	81.9	6.35	0.87	77.87	11.04	0.74
2	M	77.65	21.89	0.83	82.32	7.9	0.93
3	F	217	5.74	0.9	248.8	−19.29	0.88
4	F	101.1	−1.34	0.91	99.28	−8.31	0.71
5	M	145.4	34.59	0.8	136.6	38.62	0.71
6	M	76.28	8.41	0.83	55.26	18.03	0.71

Abbreviations: EMG RMS, electromyography root mean square; F, female; M, male.

* *p* < 0.001 for all subjects.

If assuming the beginning of the profiles (time ~0 s) as a reference of resting muscle, *c*_55_ was 12.83 ± 7.14 kPa (ranged from 3.89 and 22.81 kPa) and 11.77 ± 6.44 kPa (ranged from 2.98 and 18.66 kPa), in the first and second series, respectively. On the other hand, the corresponding values of the plateau (time between 20 and 25 s) were 113.35 ± 44.06 kPa (ranged from 65.72 and 187.04 kPa) in the first series and 98.87 ± 32.20 kPa (ranged from 65.99 and 192.21 kPa) in the second.

Regarding the repeatability of the measurements, Table 3 shows the statistical analysis results according to the two intensity levels of isometric contraction (0.3 and 0.7 of the maximal EMG RMS activity). In this way, the high ICC, low SEM, and the CV obtained in each condition indicate a very good reproducibility of the estimations. Concerning the comparison of $c_{55}$ estimated at both isometric contraction levels, a significant main effect of the contraction intensity (*p* = 0.000) was found. This implies that $c_{55}$ estimated at 0.7 of the maximal EMG RMS activity was significantly higher than at 0.3. On the other hand, this main effect was not found when comparing the estimates obtained from the two series of trapezoidal profiles, indicating that *c*_55_ was not significantly different (*p* > 0.05) between them.

**TABLE 3:** Average values of *c*_55_ estimated at two levels of contraction intensity and results of repeatability analysis. The *c*_55_ was calculated for each subject in both series from the regressions coefficients of Table 2 and then averaged (the standard deviation is shown between brackets)

	EMG RMS (relative to max.)	
	0.3	0.7
c_55_ (kPa) First series	47.57 (21.78)	94.20 (41.76)
c_55_ (kPa) Second series	43.00 (21.01)	89.67 (43.11)
ICC	0.91	0.99
SEM CV (%)	6.17 13.60	4.05 4.41

Abbreviations: CV, coefficient of variation; EMG RMS, electromyography root mean square.

## 4 DISCUSSION

The main purpose of the present work was to test the performance of a new method able to correlate the surface wave propagation with the *c*_55_ of the skeletal muscle. The study showed that the NU‐SWE could reliably estimate the tissue elasticity and assess its relationship regarding other biomechanical properties of muscle. In this sense, the results of *experiments 1* and *2* agreed qualitatively and quantitatively to those of previous works using ultrasound elastography.

**FIGURE 5:**
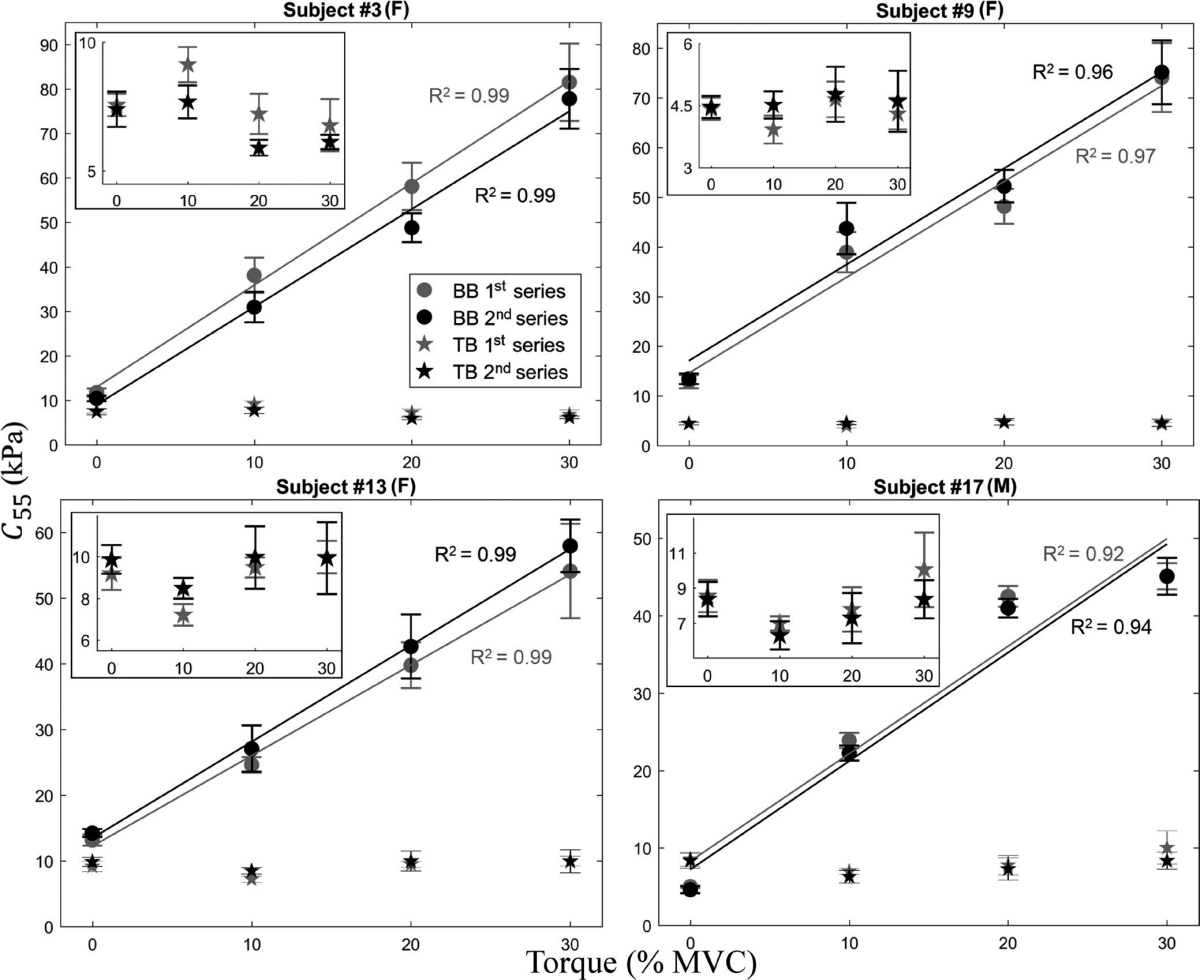
*c*_55_ versus torque in the biceps brachii (BB) and triceps brachii (TB) of four representative subjects during *experiment 1*. The error bar represents the standard deviation over ten measurements. Solid lines show the linear fit performed from the averaged values. The inset in each figure is a magnification of the results in TB, added for better visualization. F, female; M, male

Regarding the results of *experiment 1*, the *c*_55_ measured in the BB and TB muscles at rest were ~10 kPa (Figure 5; Table 1). The lowest values were 5.57 ± 1.05 and 4.42 ± 0.27 kPa, respectively, which are in very good agreement with previous works using the ultrasonic methods since the reported values ranging from 3.11 ± 0.42 kPa (BB) and 3.05 ± 0.52 kPa (TB) (Lacourpaille et al., 2012). Likewise, both the average rest values and their range of variation were also comparable to those obtained in previous studies carried out in vivo with magnetic resonance elastography and SSI (~10–20 kPa; Bouillard, Nordez, et al., 2012; Jenkyn et al., 2003; Lapole et al., 2015; Nordez & Hug, 2010; Uffmann et al., 2004; Yoshitake et al., 2014), as well as in vitro performing a stress‐strain curve (~10 kPa; Lieber, 2002). As the most accurate comparison is made in resting conditions, since the contraction intensity can vary between different studies (Nordez & Hug, 2010), these results show the good agreement between NU‐SWE and reference elastography methods. On the other hand, the behavior between 10% to 30% of MVC was opposite for both muscles. While in the TB the muscle shear elasticity did not differ significantly compared to the muscle at rest, in the BB the elasticity was greater as the torque level increased (Figure 5; Table 1). These results are also in good agreement with previous studies using SSI. In this regard, Nordez & Hug (2010) and Bouillard, Nordez, et al., 2012) reported values from 15–30, 30–60, and 45–70 kPa, for 10%, 20%, and 30% of MVC, respectively. Likewise, Yoshitake et al. (2014) and Lapole et al. (2015) reported values of 45–60, 70–90, and 95–115 kPa, for the same conditions of contraction intensity. Although, as indicated above, the comparisons in such conditions are more difficult to establish than in resting muscle, our estimates are also comparable since they are in the range of the variation found in such works. This variation in the shear modulus of BB during different intensities of isometric contraction could be partially explained by individual differences in load sharing between muscles (Bouillard, Nordez, et al., 2012; Rengifo et al., 2010). Beyond the above, our results in TB and BB muscles are consistent regarding their function during isometric contraction, since they act as extensor and flexor muscles of the forearm, respectively. These results reinforce the evidence in favor that a systematic increase in EMGs amplitude of antagonist muscles (i.e., increased coactivation) at high force levels can be partially explained by crosstalk than increased drive to the antagonist muscles (Hug et al., 2015; Solomonow et al., 1994). However, some coactivation can be seen in some people for mechanical purposes to stabilize the elbow joint (Le et al., 2017; Riemann & Lephart, 2002). Overall, these observations suggest that elastography might provide a unique opportunity to reconsider our current understanding of muscle co‐contraction (Avrillon et al., 2018; Hug et al., 2015).

In *experiment 2*, the results showed that the NU‐SWE combined with the EMG allowed to effectively assess the changes of the elasticity and EMGs activity of the muscle because of the increased elbow flexion torque. As is shown in Table 2, a considerable variation was found in the slopes between *c*_55_ and the EMG activity level of *experiment 2* (the ratio between the highest and lowest slope was 2.84). This observation agreed with a previous study using SSI and EMGs (Nordez & Hug, 2010), where a ratio of 2.72 was also found in the BB. Thus, it seems to be a variable feature among subjects, derived from the intrinsic properties of the muscular system. In this sense, as in *experiment 1* and based on previous works (Avrillon et al., 2018; Bouillard, Nordez, et al., 2012; Hug et al., 2015; Rengifo et al., 2010), we suggest that differences in load sharing and muscle co‐contraction could provide a possible explanation for this phenomenon. In addition to the above, it is also important to note that a hysteresis behavior was observed in some subjects for both *c*_55_ and EMG activity (e.g., subject #3, Figure 3). Besides, in some subjects, the trapezoidal profiles of such variables showed occasional mismatches within the same series (e.g., subject #2, Figure 3). The above can be partially due to differences in the execution of the protocol, since the recordings were not performed simultaneously to avoid the contamination of the EMG signals by the surface vibration artifacts. This may explain the lower values of *R* ^2^~ 0.7 (still significant, *p* < 0.001) observed in specific cases.

Comparing the estimates obtained between 0% and 40% of MVC, the results of *experiment 2* were analogous to those of previous works. Regarding the resting values, our estimates at the beginning (*t*~ 0 s) of the profiles agree with the corresponding values of *experiment 1*. Also, these are in complete agreement with those of the literature, where values between ~ 4 – 20 kPa were reported by using the reference elastography and rheological methods (Bouillard, Nordez, et al., 2012; Creze et al., 2018; Jenkyn et al., 2003; Lacourpaille et al., 2012; Lapole et al., 2015; Lieber, 2002; Nordez & Hug, 2010; Uffmann et al., 2004; Yoshitake et al., 2014). For the 40% of MVC, the literature reports values for BB ranging from ~65 to 80 kPa (Bouillard, Nordez, et al., 2012; Nordez & Hug, 2010), as well as ~100 to 160 kPa (Lapole et al., 2015; Yoshitake et al., 2014). Since these measurements were performed at a moderate intensity contraction level, the comparisons are quite difficult to establish, as we discussed above. Nevertheless, our results also showed a good agreement, since during the plateau phase (40% of MVC) they were within the same range of variation depicted in such studies. Additionally, the intermediate values measured during the ascending phase between 10 and 30 of MVC (~ 20 – 100 kPa, see data between 5 and 15 s in Tables S4 and S6), were also very similar to those of *experiment 1* and references (Bouillard, Nordez, et al., 2012; Lapole et al., 2015; Nordez & Hug, 2010; Yoshitake et al., 2014). Concerning the estimate's reliability, the results are in complete agreement to those of previous works using the ultrasound elastography, where ICC, CV, and SEM values around 0.9, 10%, 4 kPa were estimated, respectively (Lapole et al., 2015; Nordez & Hug, 2010). In this sense, the NU‐SWE has demonstrated high reliability to infer the EMG activity level. Thus, the results obtained in this experiment, reinforce the results of *experiment 1* regarding the certainty of the NU‐SWE estimates. They also highlight that it can be used together with other methods to study different aspects of muscle biomechanics.

Another relevant fact that emerges from *experiments 1* and *2* is that the linear model can be a good approximation to explain the relation of *c*_55_ with the elbow flexion torque and EMG activity during BB's isometric flexion. This was previously studied in different muscles (e.g., BB, first dorsal interosseous, abductor digiti minimi, gastrocnemius medialis, tibialis anterior) by using ultrasound elastography during passive stretching and isometric contractions, even in conditions of neuromuscular fatigue (Ateş et al., 2015; Bouillard, Hug, et al., 2012; Bouillard et al., ,^2011^; Koo et al., 2013, 2014; Lapole et al., 2015; Sasaki et al., 2014; Yoshitake et al., 2014). Nevertheless, the non‐linear relationship between the EMG activity and joint torque has been frequently reported for large muscles such as BB (Lawrence & De Luca, 1983; Nordez & Hug, 2010). In addition, Bouillard, Nordez, et al. (2012) and Nordez & Hug (2010) show that the characteristic shape of the relation between the BB elasticity and joint torque is more quadratic than linear. It consists of a little change initially (~ 0% – 10% of MVC), but a rapid increment in the elasticity as the torque increases. In this sense, Bouillard, Nordez, et al. (2012) gives second‐order polynomial equations that fit the experimental data with an *R* ^2^ = 0.99. Nevertheless, a linear fit of these data provides coefficients of determination ~0.9, very similar to those of the present study and the references (Yoshitake et al., 2014) and (Lapole et al., 2015). Therefore, we believe that the results of *experiment 1* do not contradict the non‐linear behavior described by previous studies (Bouillard, Nordez, et al., 2012; Nordez & Hug, 2010), but on the contrary, they are very similar. Task specificity and differences in motor control may partially explain the differences (Lapole et al., 2015), but these can be explained mainly based on methodological aspects. While in *experiment 1*, the elasticity was estimated at four discrete torque levels within the range 0% – 30% of MVC, several intermediate values were collected in Bouillard, Nordez, et al. (2012) and Nordez et al. (2010). In this way, since in *experiment 2 c*_55_ was collected with a higher sample frequency, the results had a better resolution. Thus, it was possible to characterize the curvilinear relation of *c*_55_ and EMG activity level regarding the elbow flexion torque, for both ascending and descending phases of the trapezoidal profiles (especially between 0% and 10% of MVC). Beyond these considerations, our results agree with those of recent works, when suggesting the validity of the linear model to simplify future research concerning the changes of these variables according to the isometric contraction intensity (Lapole et al., 2015; Yoshitake et al., 2014).

The aim of the present study was not to validate the NU‐SWE but to show its performance, reliability, and feasibility to estimate in vivo and real‐time the muscle shear elasticity. Therefore, the experiments we carried out in this work did not include a direct comparison between the NU‐SWE and another known elastography method. Nevertheless, in previous works, we have performed such comparisons in phantoms and beef samples regarding the ultrasound shear wave elastography (Benech et al., ,^2019^, ^2021^; Grinspan et al., 2016). These studies showed that both approaches provide comparable results, especially within the range of frequencies employed in this work. Beyond the above, further validation in skeletal muscle is still needed. Unlike previous works in skeletal muscle using SSI (Bouillard, Hug, et al., 2012; Bouillard, Nordez, et al., ,^2011^, ^2012^; Nordez & Hug, 2010; Yoshitake et al., 2014), this method does not impose a saturation limit on the measurement of *c*_55_. In this sense, as the modern versions of SSI, the NU‐SWE could be potentially able to characterize a broad range of muscle shear elasticity values. Further studies are needed to assess the capability of the NU‐SWE to measure this parameter at MVC, which would be very advantageous compared to most ultrasound elastography techniques.

Due to its low spatial resolution, the NU‐SWE cannot locally map the tissue elasticity as the reference ultrasonic methods do. It also cannot measure the elasticity of deep muscles, so its use is limited to superficial muscles. This is because at low frequencies (~100 Hz), the penetration depth of surface wave is limited to one wavelength, which is about a few centimeters since typical values of the shear velocity in soft tissues are *V*_T_~1–10 m s^−1^. In this way, the NU‐SWE can retrieve, in vivo and non‐invasively, a reliable value of the mean bulk tissue elasticity in the near‐surface below the sensors. However, in this regard, the presence of subcutaneous adipose tissue should be considered. Depending on the amount of this tissue, the estimates could be affected since the medium becomes more attenuating, making the signal/noise ratio poorer. Besides, as the presence of this tissue increases, the medium becomes softer on average. Thus, the *c*_55_ as well as the torque versus elasticity relation may not be representative of the assessed muscle. Since the subjects who participated in the present study had normal average values of body mass index (BMI; 22.71 ± 2.79) and body fat percentage (BF; 21.06 ± 4.98), it is highly unlikely that this factor may have influenced the results. Nevertheless, further studies are needed to establish with greater precision the limits imposed by the adipose tissue in the measurements, as well as to develop strategies to correct this issue when necessary.

Beyond the above, our results suggest that when their limitations can be ignored, the NU‐SWE could be suitable to extend elastography applications in muscle biomechanics. Its advantageous features are similar to the low‐frequency method recently proposed by Martin et al. (2018) to measure elasticity in tendons. However, the Timoshenko beam model employed in such work does not apply to the skeletal muscle (Timoshenko, 1921, 1922). Likewise, other low‐frequency methods proposed to measure muscle shear elasticity do not consider the incidence of the diffraction, guided wave, and near‐field effects (Benech et al., 2012; Courage, 2003; Sabra et al., 2007; Salman & Sabra, 2013; Zhang, 2016). In this sense, the new inversion algorithm of the NU‐SWE can automatically correct the biases introduced for those effects, allowing reliable and robust estimates.

It is important to point out that the present study was carried out using an initial prototype of NU‐SWE, designed just to test the method and address the objectives of *experiments 1* and *2*. We do not expect that such a prototype can be suitable to be applied outside the laboratory. Regarding the above, we are currently working on a portable version of NU‐SWE, able to be used in field measurements. In addition, we think that with further development, this method could be suitable to measure the elasticity in more than one muscle simultaneously. This is also unfeasible with the current elastography methods. In this sense, the NU‐SWE could be a useful method to deepen the research about muscle co‐contraction and the interpersonal differences in load sharing. Likewise, it could be used to estimate the individual forces exerted by superficial muscles. In this regard, recent works using SSI have begun to consider the muscle shear elasticity as an informative parameter for such a purpose (Bouillard, Hug, et al., 2012; Bouillard, Nordez, et al., 2011). Concerning its practical applications, the NU‐SWE could be a low‐cost and reliable tool to monitor the rehabilitation process after a muscle injury, as well as to assess the muscle mechanical properties in athletes as a consequence of a training plan (Creze et al., 2018; Sarto et al., 2021).

Based on all the above considerations, we think that this work constitutes the first step to extend the practical and research applications of muscle elastography. In this way, our future work will focus on improving the current prototype to make the NU‐SWE a versatile method, with potential applications in muscle biomechanics and related fields.

## 5 CONCLUSIONS

In this work, we have shown that the NU‐SWE can estimate the muscle shear elasticity in a comparable way to the reference elastography methods. In this regard, the results concerning *experiment 1* (muscle shear elasticity vs. elbow flexion torque) and *2* (muscle shear elasticity vs. EMG activity level) were quantitatively and qualitatively in good agreement with previous works using ultrasound elastography. The latter was largely due to the new inversion algorithm on which the method is based. This is its main innovation regarding other low‐frequency elastography methods described in the literature. Unlike these, the NU‐SWE can correct the shear wave velocity value of the biases introduced by the diffraction, guided wave, and near field effects. The main disadvantages of the method are that it cannot construct an elasticity map of the tissue, and their use is limited to the superficial muscles. However, it provides many advantageous features, such as being wearable, transportable, easy handle, and potentially able to be used in more than one muscle simultaneously. Besides, the estimates are performed in vivo, non‐invasively, and in real‐time. Thus, the NU‐SWE method offers the possibility to extend the practical and research applications of elastography in muscle biomechanics and related fields. This could be useful to deepen the knowledge about the mechanical behavior of the muscle, especially in those areas where the use of existing methods is not feasible or possible.

## DISCLOSURES

The NU‐SWE method presented in this article was included in a PCT patent application, presented by Universidad de la República (UdelaR) as patent applicant and assignee (inventors: Nicolás Benech, Gustavo Grinspan, Sofía Aguiar, Carlos Negreira). PCT patent application is currently in National Phase and under examination in different territories. The patent application was licensed for the meat industry but is open for licensing for other fields.

## AUTHOR CONTRIBUTION

Gustavo A. Grinspan, Hélio V. Cabral, Leonardo M. L. de Souza, Liliam F. de Oliveira, and Nicolás Benech: conception and design of the research; Gustavo A. Grinspan, Hélio V. Cabral, and Leonardo M. L. de Souza: performed experiments and acquired data; Gustavo A. Grinspan, Hélio V. Cabral, Leonardo M. L. de Souza, Liliam F. de Oliveira, SofíaAguiar, Ernesto Blanco, and Nicolás Benech: analyzed and interpreted data; Gustavo A. Grinspan, Hélio V. Cabral, and Nicolás Benech: prepared figures; Gustavo A. Grinspan, Hélio V. Cabral, and Nicolás Benech: drafted manuscript; Gustavo A. Grinspan, Hélio V. Cabral, Leonardo M. L. de Souza, Liliam F. de Oliveira, SofíaAguiar, Ernesto Blanco, and Nicolás Benech: revised the manuscript critically for intellectual content; Gustavo A. Grinspan, Hélio V. Cabral, Leonardo M. L. de Souza, Liliam F. de Oliveira, SofíaAguiar, Ernesto Blanco, and Nicolás Benech: approved the final version of the submitted manuscript.

## Supporting information



Fig S1‐4

Table S1‐10

## Figures and Tables

**FIGURE A1:**
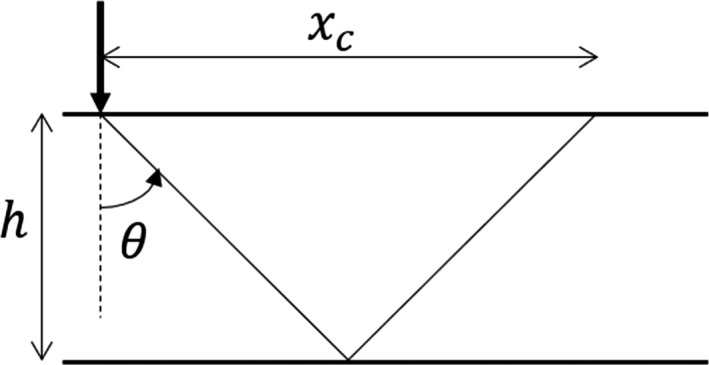
Directivity pattern of the shear wave in a medium with depth *h*. The distance *x*_c_ represents the near‐field zone where only surface waves propagate. Within this zone, the dispersion curve is due to interference between Rayleigh and leaky surface wave.

## Data Availability

The data that support the findings of this study are available from the corresponding author upon reasonable request from a qualified researcher.

## APPENDIX A

### Signal processing algorithm

This algorithm estimates the phase shift (∆*ϕ*) between the signals recorded by the linear array of vibration sensors. A band‐pass filter centered on the source's frequency with a 50 % bandwidth is applied to raw signals to eliminate unwanted frequencies (Figures 1c,d). Then, the phase shift between the recorded signals is computed at a selected frequency within the bandwidth by Fourier transform. The phase shift is converted to time delay by dividing it between the corresponding angular frequency (*ω*_0_). Finally, the phase velocity (*V*) is computed as follows:

(A1)
$$V=\frac{\omega_{0}d^{\prime }}{\Delta \phi },$$

where *d*′ is the distance between sensors.

### Inversion algorithm to correct the incidence of the guided wave propagation and near field effects

Expression (A1) is used to compute the phase velocity for each frequency within the bandwidth of the signal. After that, a dispersion curve *V*(*ω*) is obtained. As explained in (Benech et al., 2017), most of the wave energy in the bulk of soft tissues propagates as shear waves in the low‐frequency limit. Due to the large difference between the speed of compressional and shear waves, there is no mode conversion in boundary reflection. Thus, the shear wave reflects back as a shear wave. Besides, the directivity pattern of the shear wave makes an angle *θ* with the normal (Figure A1).

Therefore, there exists a distance *x*_c_ where only surface waves propagate given by:

(A2)
$$x_{c}=2h\tan\left(\theta \right).$$

As shown in (Benech et al., 2017), if the wavefield is measured at a distance $x\gg x_{c}$ from the source, the dispersion curve *V*(*ω*) fits the model of Rayleigh‐Lamb. However, if *x* ≤ *x*_c_ the dispersion curve is due to interference between the Rayleigh surface wave and the leaky surface wave. For transversally isotropic solids, like skeletal muscle, $\theta ≅60^{\circ }$. The mean depth in BB is *h* = 3.5 cm and for TB is *h* = 2.5 cm. Therefore, the distance $x_{c}≅12$ and 9 cm, respectively. Thus, the NU‐SWE acquires data in the near‐field zone. The vertical component *u_z_* (*x*, *t*) of the displacement field in the surface of the tissue is given by:

(A3)
$$u_{z}\left(x,\;t\right)=A_{R}\left[e^{‐ikx}+A_{L}e^{‐\zeta x}e^{‐iqx}\right]e^{i\omega t},$$

where *A*_R_ is the amplitude and *k* the wave number of the Rayleigh wave, *A*_L_ is the relative amplitude between the leaky wave and the Rayleigh wave, ζ and *q* are the imaginary and real parts of the leaky wave wavenumber, respectively. The phase *ϕ*(*x*) of the quantity between brackets is *ϕ*(*x*) = *a*tan (*N*(*x*)/*D*(*x*)) where:

(A4)
$$N\left(x\right)=‐\left[\sin\left(kx\right)+A_{L}e^{‐\zeta x}\sin\left(qx\right)\right],$$

(A5)
$$D\left(x\right)=\cos\left(kx\right)+A_{L}e^{‐\zeta x}\cos\left(qx\right).$$

The phase speed is, therefore:

(A6)
$$V\left(\omega \right)=\omega {\left(\frac{\partial \phi }{\partial x}\right)}^{‐1}=\omega \left[\frac{D^{2}+N^{2}}{N^{\prime }D‐ND^{\prime }}\right],$$

where the prime indicates derivative with respect to x. Finally, to retrieve the shear wave speed, the experimental data is fitted in the least square sense to the equation above.

## References

[phy214955-bib-0001] Ateş, F. , Hug, F. , Bouillard, K. , Jubeau, M. , Frappart, T. , Couade, M. , Bercoff, J. , & Nordez, A. (2015). Muscle shear elastic modulus is linearly related to muscle torque over the entire range of isometric contraction intensity. Journal of Electromyography and Kinesiology, 25(4), 703–708. 10.1016/j.jelekin.2015.02.005 25956546

[phy214955-bib-0002] Avrillon, S. , Hug, F. , & Guilhem, G. (2018). Between‐muscle differences in coactivation assessed using elastography. Journal of Electromyography and Kinesiology, 43, 88–94. 10.1016/j.jelekin.2018.09.007 30265870

[phy214955-bib-0003] Benech, N. , Aguiar, S. , & Grinspan, G. A. (2021). Monitoring ageing in beef samples using surface wave elastography: A feasibility study. Journal of Food Engineering, 307, 110647. 10.1016/j.jfoodeng.2021.110647

[phy214955-bib-0004] Benech, N. , Aguiar, S. , Grinspan, G. A. , Brum, J. , & Negreira, C. (2012). In vivo assessment of muscle mechanical properties using a low‐cost surface wave method. 2012 IEEE International Ultrasonics Symposium, 2571–2574. 10.1109/ULTSYM.2012.0644

[phy214955-bib-0005] Benech, N. , Brum, J. , Grinspan, G. A. , Aguiar, S. , & Negreira, C. A. (2017). Analysis of the transient surface wave propagation in soft‐solid elastic plates. Journal of the Acoustical Society of America, 142(5), 2919–2932. 10.1121/1.4993633 29195471

[phy214955-bib-0006] Benech, N. , Grinspan, G. A. , Aguiar, S. , & Negreira, C. A. (2019). Surface wave elastography: Device and method. Measurement Science & Technology, 30(3), 035701. 10.1121/1.4993633

[phy214955-bib-0007] Bercoff, J. , Tanter, M. , & Fink, M. (2004). Supersonic shear imaging: A new technique for soft tissue elasticity mapping. IEEE Transactions on Ultrasonics, Ferroelectrics, and Frequency Control, 51(4), 396–409. 10.1109/tuffc.2004.1295425 15139541

[phy214955-bib-0008] Bouillard, K. , Hug, F. , Guével, A. , & Nordez, A. (2012). Shear elastic modulus can be used to estimate an index of individual muscle force during a submaximal isometric fatiguing contraction. Journal of Applied Physiology, 113(9), 1353–1361. 10.1152/japplphysiol.00858.2012 22984244

[phy214955-bib-0009] Bouillard, K. , Nordez, A. , Hodges, P. W. , Cornu, C. , & Hug, F. (2012). Evidence of changes in load sharing during isometric elbow flexion with ramped torque. Journal of Biomechanics, 45(8), 1424–1429. 10.1016/j.jbiomech.2012.02.020 22406469

[phy214955-bib-0010] Bouillard, K. , Nordez, A. , & Hug, F. (2011). Estimation of individual muscle force using elastography. PLoS One, 6(12), e29261. 10.1371/journal.pone.0029261 22229057 PMC3244452

[phy214955-bib-0011] Catheline, S. , Gennisson, J. L. , Delon, G. , Fink, M. , Sinkus, R. , Abouelkaram, S. , & Culioli, J. (2004). Measuring of viscoelastic properties of homogeneous soft solid using transient elastography: An inverse problem approach. Journal of the Acoustical Society of America, 116(6), 3734–3741. 10.1121/1.1815075 15658723

[phy214955-bib-0012] Catheline, S. , Wu, F. , & Fink, M. (1999). A solution to diffraction biases in sonoelasticity: The acoustic impulse technique. Journal of the Acoustical Society of America, 105(5), 2941–2950. 10.1121/1.426907 10335643

[phy214955-bib-0013] Courage, W. (2003). Measuring device for measuring the elastic properties of a surface structure (US patent no. 6,619,423). U.S. Patent and Trademark Office.

[phy214955-bib-0014] Creze, M. , Nordez, A. , Soubeyrand, M. , Rocher, L. , Maître, X. , & Bellin, M. F. (2018). Shear wave sonoelastography of skeletal muscle: Basic principles, biomechanical concepts, clinical applications, and future perspectives. Skeletal Radiology, 47(4), 457–471. 10.1007/s00256-017-2843-y 29224123

[phy214955-bib-0015] Deffieux, T. , Montaldo, G. , Tanter, M. , & Fink, M. (2009). Shear wave spectroscopy for in vivo quantification of human soft tissues visco‐elasticity. IEEE Transactions on Medical Imaging, 28(3), 313–322. 10.1109/TMI.2008.925077 19244004

[phy214955-bib-0016] Gennisson, J. L. , Catheline, S. , Chaffaı, S. , & Fink, M. (2003). Transient elastography in anisotropic medium: Application to the measurement of slow and fast shear wave speeds in muscles. Journal of the Acoustical Society of America, 114(1), 536–541. 10.1121/1.1579008 12880065

[phy214955-bib-0017] Gennisson, J. L. , Cornu, C. , Catheline, S. , Fink, M. , & Portero, P. (2005). Human muscle hardness assessment during incremental isometric contraction using transient elastography. Journal of Biomechanics, 38(7), 1543–1550. 10.1016/j.jbiomech.2004.07.013 15922766

[phy214955-bib-0018] Gennisson, J. L. , Deffieux, T. , Macé, E. , Montaldo, G. , Fink, M. , & Tanter, M. (2010). Viscoelastic and anisotropic mechanical properties of in vivo muscle tissue assessed by supersonic shear imaging. Ultrasound in Medicine and Biology, 36(5), 789–801. 10.1016/j.ultrasmedbio.2010.02.013 20420970

[phy214955-bib-0019] Grinspan, G. A. , Aguiar, S. , & Benech, N. (2016). Optimization of a surface wave elastography method through diffraction and guided waves effects characterization. Journal of Physics: Conference Series, 705(2016), 012014. 10.1088/1742-6596/705/1/012014

[phy214955-bib-0020] Hammer, Ø. , Harper, D. A. T. , & Ryan, P. D. (2001). PAST: Paleontological statistics software package for education and data analysis. Palaeontologica Electron, 4(1), 1–9.

[phy214955-bib-0021] Heers, G. , Jenkyn, T. , Dresner, M. A. , Klein, M. O. , Basford, J. R. , Kaufman, K. R. , Ehman, R. L. , & An, K. N. (2003). Measurement of muscle activity with magnetic resonance elastography. Clinical Biomechanics, 18(6), 537–542. 10.1016/S0268-0033(03)00070-6 12828903

[phy214955-bib-0022] Hopkins, W. G. (2000). Measures of reliability in sports medicine and science. Sports Medicine, 30(1), 1–15. 10.2165/00007256-200030010-00001 10907753

[phy214955-bib-0023] Hug, F. , Tucker, K. , Gennisson, J. L. , Tanter, M. , & Nordez, A. (2015). Elastography for muscle biomechanics: Toward the estimation of individual muscle force. Exercise and Sport Sciences Reviews, 43(3), 125–133. 10.1249/JES.0000000000000049 25906424

[phy214955-bib-0024] Jenkyn, T. R. , Ehman, R. L. , & An, K. N. (2003). Noninvasive muscle tension measurement using the novel technique of magnetic resonance elastography (MRE). Journal of Biomechanics, 36(12), 1917–1921. 10.1016/S0021-9290(03)00005-8 14614945

[phy214955-bib-0025] Koo, T. K. , Guo, J. Y. , Cohen, J. H. , & Parker, K. J. (2013). Relationship between shear elastic modulus and passive muscle force: An ex‐vivo study. Journal of Biomechanics, 46(12), 2053–2059. 10.1016/j.jbiomech.2013.05.016 23769175

[phy214955-bib-0026] Koo, T. K. , Guo, J. Y. , Cohen, J. H. , & Parker, K. J. (2014). Quantifying the passive stretching response of human tibialis anterior muscle using shear wave elastography. Clinical Biomechanics, 29(1), 33–39. 10.1016/j.clinbiomech.2013.11.009 24295566

[phy214955-bib-0027] Lacourpaille, L. , Hug, F. , Bouillard, K. , Hogrel, J. Y. , & Nordez, A. (2012). Supersonic shear imaging provides a reliable measurement of resting muscle shear elastic modulus. Physiological Measurement, 33(3), N19–N28. 10.1088/0967-3334/33/3/N19 22370174

[phy214955-bib-0028] Lapole, T. , Tindel, J. , Galy, R. , & Nordez, A. (2015). Contracting biceps brachii elastic properties can be reliably characterized using supersonic shear imaging. European Journal of Applied Physiology, 115(3), 497–505. 10.1007/s00421-014-3037-0 25366254

[phy214955-bib-0029] Lawrence, J. H. , & De Luca, C. J. (1983). Myoelectric signal versus force relationship in different human muscles. Journal of Applied Physiology, 54(6), 1653–1659. 10.1152/jappl.1983.54.6.1653 6874489

[phy214955-bib-0030] Le, P. , Best, T. M. , Khan, S. N. , Mendel, E. , & Marras, W. S. (2017). A review of methods to assess coactivation in the spine. Journal of Electromyography and Kinesiology, 32, 51–60. 10.1016/j.jelekin.2016.12.004 28039769

[phy214955-bib-0031] Lieber, R. L. (2002). Skeletal muscle structure, function and plasticity. Lippincott Willians & Wilkins.

[phy214955-bib-0032] Martin, J. A. , Brandon, S. C. E. , Keuler, E. M. , Hermus, J. R. , Ehlers, A. C. , & Segalman, D. J. (2018). Gauging force by tapping tendons. Nature Communications, 9(1), 1592. 10.1038/s41467-018-03797-6 PMC5913259 29686281

[phy214955-bib-0033] Nishihara, K. , Kawai, H. , Chiba, Y. , Kanemura, N. , & Gomi, T. (2013). Investigation of innervation zone shift with continuous dynamic muscle contraction. Computational and Mathematical Methods in Medicine, 2013, 1–7. 10.1155/2013/174342 PMC3677009 23762179

[phy214955-bib-0034] Nordez, A. , Gennisson, J. L. , Casari, P. , Catheline, S. , & Cornu, C. (2008). Characterization of muscle belly elastic properties during passive stretching using transient elastography. Journal of Biomechanics, 41(10), 2305–2311. 10.1016/j.jbiomech.2008.03.033 18539284

[phy214955-bib-0035] Nordez, A. , Guével, A. , Casari, P. , Catheline, S. , & Cornu, C. (2009). Assessment of muscle hardness changes induced by a submaximal fatiguing isometric contraction. Journal of Electromyography and Kinesiology, 19(3), 484–491. 10.1016/j.jelekin.2007.11.005 18158253

[phy214955-bib-0036] Nordez, A. , & Hug, F. (2010). Muscle shear elastic modulus measured using supersonic shear imaging is highly related to muscle activity level. Journal of Applied Physiology, 108(5), 1389–1394. 10.1152/japplphysiol.01323.2009 20167669

[phy214955-bib-0037] Pitre Jr., J. J. , Kirby, M. A. , Gao, L. , Li, D. S. , Shen, T. , Wang, R. K. , O'Donnell, M. , & Pelivanov, I. (2019). Super‐shear evanescent waves for non‐contact elastography of soft tissues. Applied Physics Letters, 115(8), 083701. 10.1063/1.5111952 32127722 PMC7043857

[phy214955-bib-0038] Rainoldi, A. , Melchiorri, G. , & Caruso, I. (2004). A method for positioning electrodes during surface EMG recordings in lower limb muscles. Journal of Neuroscience Methods, 134(1), 37–43. 10.1016/j.jneumeth.2003.10.014 15102501

[phy214955-bib-0039] Rengifo, C. , Aoustin, Y. , Plestan, F. , & Chevallereau, C. (2010). Distribution of forces between synergistics and antagonistics muscles using an optimization criterion depending on muscle contraction behavior. Journal of Biomechanical Engineering, 132(4), 041009. 10.1115/1.4001116 20387972

[phy214955-bib-0040] Riemann, B. L. , & Lephart, S. M. (2002). The sensorimotor system, Part II: The role of proprioception in motor control and functional joint stability. Journal of Athletic Training, 37(1), 80. 16558671 PMC164312

[phy214955-bib-0041] Sabra, K. G. , Conti, S. , Roux, P. , & Kuperman, W. A. (2007). Passive in vivo elastography from skeletal muscle noise. Applied Physics Letters, 90(19), 194101–194103. 10.1063/1.2737358

[phy214955-bib-0042] Salman, M. , & Sabra, K. G. (2013). Surface wave measurements using a single continuously scanning laser doppler vibrometer: Application to elastography. Journal of the Acoustical Society of America, 133(3), 1245–1254. 10.1121/1.4789929 23463997

[phy214955-bib-0043] Sarto, F. , Spörri, J. , Fitze, D. P. , Quinlan, J. I. , Narici, M. V. , & Franchi, M. V. (2021). Implementing ultrasound imaging for the assessment of muscle and tendon properties in elite sports: Practical aspects, methodological considerations and future directions. Sports Medicine, 1–20. 10.1007/s40279-021-01436-7 33683628 PMC8124062

[phy214955-bib-0044] Sarvazyan, A. (1993). Shear acoustic properties of soft biological tissues in medical diagnostics. Journal of the Acoustical Society of America, 93(4), 2329–2330. 10.1121/1.406353

[phy214955-bib-0045] Sasaki, K. , Toyama, S. , & Ishii, N. (2014). Length‐force characteristics of in vivo human muscle reflected by supersonic shear imaging. Journal of Applied Physiology, 117(2), 153–162. 10.1152/japplphysiol.01058.2013 24876360

[phy214955-bib-0046] Solomonow, M. , Baratta, R. , Bernardi, M. , Zhou, B. , Lu, Y. , Zhu, M. , & Acierno, S. (1994). Surface and wire EMG crosstalk in neighbouring muscles. Journal of Electromyography and Kinesiology, 4(3), 131–142. 10.1016/1050-6411(94)90014-0 20870553

[phy214955-bib-0047] Tanter, M. , Bercoff, J. , Athanasiou, A. , Deffieux, T. , Gennisson, J. L. , Montaldo, G. , Muller, M. , Tardivon, A. , & Fink, M. (2008). Quantitative assessment of breast lesion viscoelasticity: Initial clinical results using supersonic shear imaging. Ultrasound in Medicine and Biology, 34(9), 1373–1386. 10.1016/j.ultrasmedbio.2008.02.002 18395961

[phy214955-bib-0048] Timoshenko, S. P. L. (1921). LXVI. On the correction for shear of the differential equation for transverse vibrations of prismatic bars. Philosophical Magazine Series 1, 41(245), 744–746. 10.1080/14786442108636264

[phy214955-bib-0049] Timoshenko, S. P. (1922). X. On the transverse vibrations of bars of uniform cross‐section. Philosophical Magazine Series 1, 43(253), 125–131. 10.1080/14786442208633855

[phy214955-bib-0050] Uffmann, K. , Maderwald, S. , Ajaj, W. , Galban, C. G. , Mateiescu, S. , Quick, H. H. , & Ladd, M. E. (2004). In vivo elasticity measurements of extremity skeletal muscle with MR elastography. NMR in Biomedicine, 17(4), 181–190. 10.1002/nbm.887 15229931

[phy214955-bib-0051] Yoshitake, Y. , Takai, Y. , Kanehisa, H. , & Shinohara, M. (2014). Muscle shear modulus measured with ultrasound shear‐wave elastography across a wide range of contraction intensity. Muscle & Nerve, 50(1), 103–113. 10.1002/mus.24104 24155045

[phy214955-bib-0052] Zhang, X. (2016). Identification of the Rayleigh surface waves for estimation of viscoelasticity using the surface wave elastography technique. Journal of the Acoustical Society of America, 140(5), 3619–3622. 10.1121/1.4966673 PMC6909991 27908086

