# Supplementary material for: Surface wave elastography is a reliable method to correlate muscle elasticity, torque, and electromyography activity level
Source: Physiol Rep. 2021 Aug 2;9(15):e14955. doi: 10.14814/phy2.14955 (PMC8326893; doi:10.14814/phy2.14955)
Supplement: Supplementary file 1 — Fig S1‐4 [file PHY2-9-e14955-s002.pdf]

Supporting Information

The following supporting figures are included for a better understanding of the experimental protocols performed in *experiments* 1 and 2.

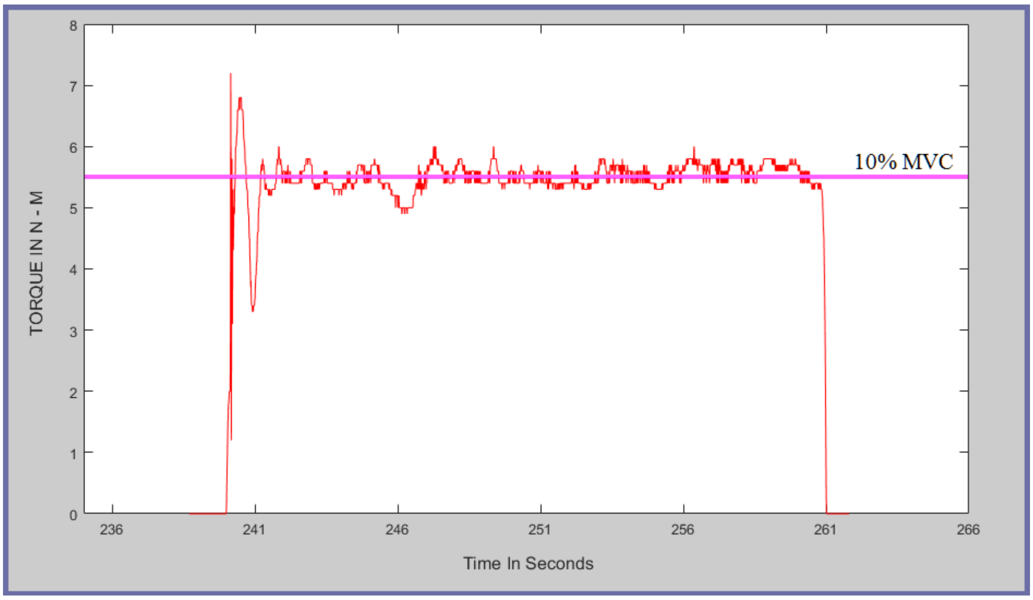

Figure SI1. Visual feedback of torque signal showing a single task of *experiment 1*. When the volunteer stabilized at the corresponding isometric contraction level, ten values of the shear elastic modulus were measured using NU-SWE.

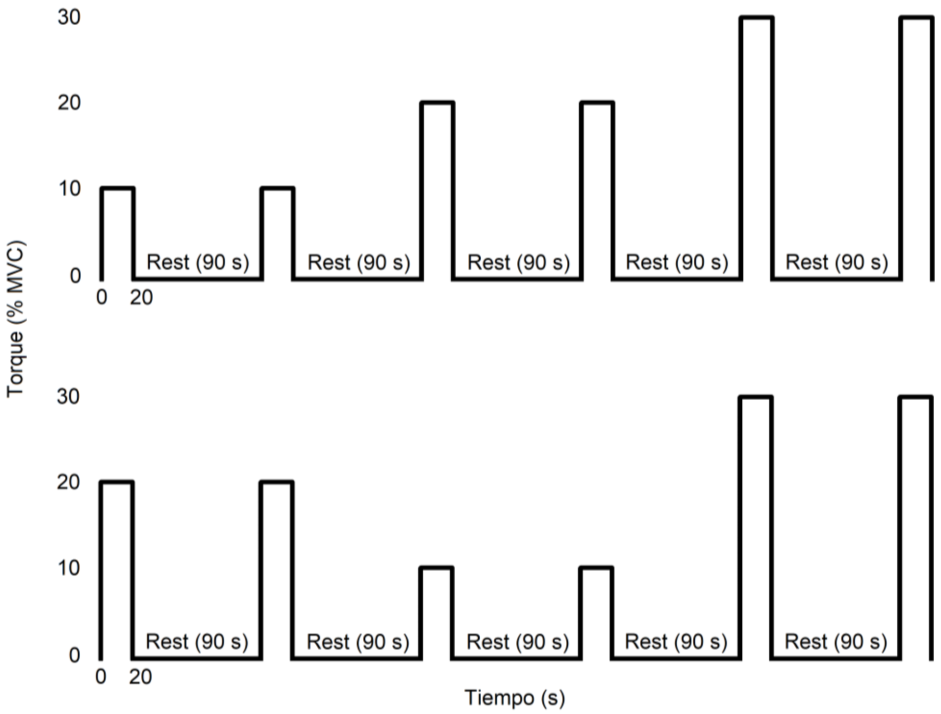

Figure SI2. Two examples of the isometric contractions corresponding to the experimental protocol of *experiment* 1. The sequences of the two series of each task are shown. The order of loads, as well as the analyzed muscle (BB or TB), was varied randomly among the volunteers to avoid bias due to the protocol.

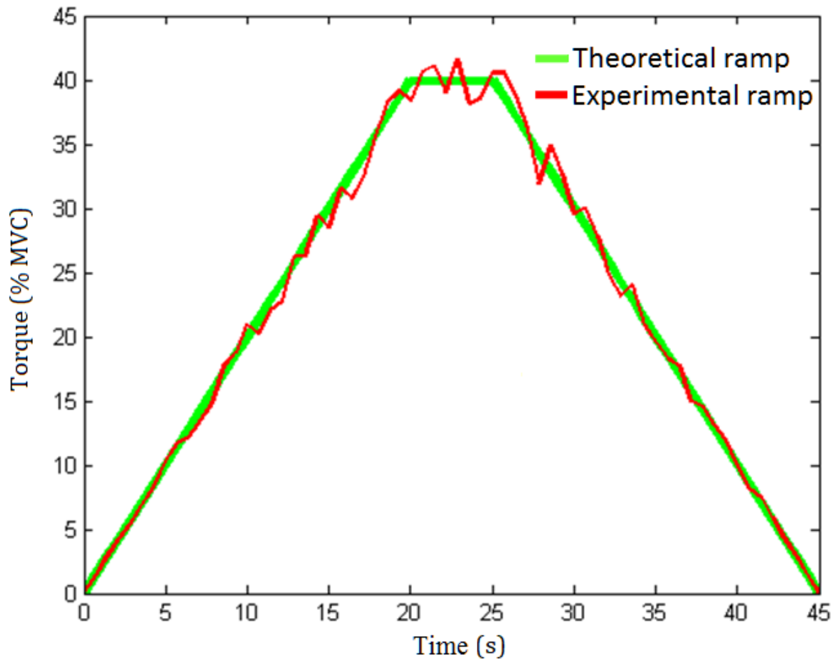

Figure SI3. Visual feedback of the trapezoidal profile employed to carry out the experimental protocol of *experiment* 2.

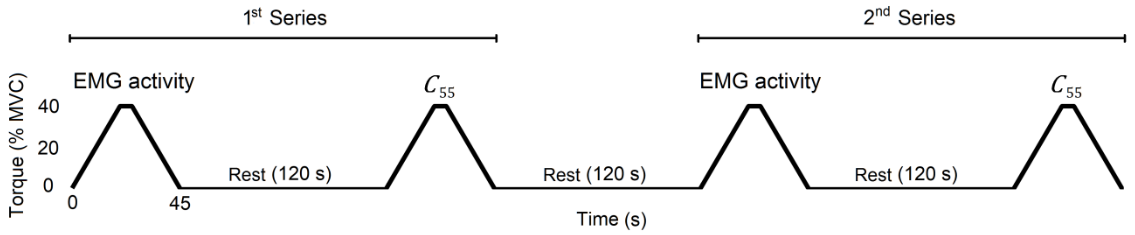

Figure SI4. Experimental protocol of *experiment* 2. The sequence shows the isometric trapezoidal profiles performed during the two series of the protocol, as well as the corresponding variable measured in each profile. The recordings of the EMG activity and the shear elastic modulus (c_{55}) were not performed simultaneously to avoid the contamination of the EMG signals by the surface vibration artifacts.
