# Supplementary material for: Surface wave elastography is a reliable method to correlate muscle elasticity, torque, and electromyography activity level
Source: Physiol Rep. 2021 Aug 2;9(15):e14955. doi: 10.14814/phy2.14955 (PMC8326893; doi:10.14814/phy2.14955)
Supplement: Supplementary file 2 — Table S1‐10 [file PHY2-9-e14955-s001.pdf]

Supplemental data:

Surface wave elastography is a reliable method to correlate muscle elasticity, torque and EMG activity level

Grinspan et al.

Table S1. Shear elastic modulus of the biceps brachii muscle obtained in both series of *experiment* 1. SD, standard deviation over ten measurements; F, female; M, male.

Biceps brachii			1st series		2nd series	
# Subject	Sex (F/M)	% MVC	c_{55} (kPa)	SD	c_{55} (kPa)	SD
1	F	0	10.91	0.92	11.23	1.49
		10	24.25	1.09	27.01	2.8
		20	46.02	8.39	52.03	8.85
		30	76.61	5.64	77.04	5.35
2	M	0	7.63	0.82	7.44	0.76
		10	30.75	3.35	26.6	0.98
		20	45.95	6.74	40.93	5.46
		30	64.2	5.14	57.19	2.03
3	F	0	11.71	0.95	10.4	0.64
		10	38.09	3.95	30.93	3.45
		20	58.09	5.33	48.79	3.21
		30	81.55	8.67	77.82	6.7
4	M	0	6.06	0.73	5.46	0.61
		10	26.32	2.09	25.42	2.04
		20	51.99	4.45	47.16	2.73
		30	109.24	8.18	101.66	9.39
5	M	0	8.25	0.49	8.86	0.63
		10	30.7	2.36	31.87	3.78
		20	38.12	2.1	43.2	5.24
		30	49.66	3.68	54.97	5.14
6	F	0	5.28	0.54	5.06	0.25
		10	37.66	1.69	37.13	4.16
		20	49.9	3.46	46.42	1.72
		30	68.31	6.75	65.28	5.04
7	M	0	5.19	0.7	4.7	0.34
		10	41.14	2.03	37.79	2.66
		20	75.17	0.89	73.96	4.37
		30	88.07	4.7	86.48	3.24
8	M	0	8.23	0.8	8.62	1.22
		10	37.47	2.73	40	2.3
		20	51.34	4.03	54.34	3.76
		30	68.8	4.84	69.6	4.51
9	F	0	12.9	1.33	13.46	1.07
		10	38.93	4.07	43.71	5.2
		20	48.15	3.51	52.2	3.27
		30	74.1	6.93	75.17	6.41

10	M	0	9.4	1.0	8.52	1.05
		10	51.37	0.88	48.45	2.37
		20	59.97	4.44	58.93	4.6
		30	91.95	3.56	83.06	7.68
11	F	0	13.05	1.05	13.11	1.44
		10	31.51	2.16	34.16	2.08
		20	40.87	4.6	53.72	4.1
		30	73.09	4.64	74.36	5.12
12	M	0	4.57	1.05	4.74	0.73
		10	25.59	5.51	26.2	1.7
		20	39.18	1.7	40.46	2.5
		30	53.94	2.25	65.0	4.35
13	F	0	13.16	0.84	14.22	0.6
		10	24.63	1.16	27.07	3.5
		20	39.77	3.48	42.63	4.89
		30	54.09	7.19	57.94	3.98
14	F	0	12.6	1.18	9.11	1.35
		10	26.43	1.93	25.04	2.56
		20	47.89	5.67	46.13	3.27
		30	60.37	6.84	55.8	5.98
15	F	0	14.53	1.65	11.69	1.44
		10	37.07	3.63	37.76	3.35
		20	49.9	5.53	52.62	4.1
		30	62.77	4.59	65.56	8.78
16	F	0	16.06	1.97	14.02	1.68
		10	28.08	2.8	27.92	2.95
		20	44.0	3.88	42.27	3.0
		30	67.89	7.79	67.17	5.92
17	M	0	5.01	0.18	4.61	0.47
		10	23.88	1.0	22.26	0.98
		20	42.5	1.32	40.99	1.2
		30	45.09	1.68	45.09	2.38
18	M	0	8.75	0.64	9.74	0.65
		10	29.63	2.51	30.11	3.5
		20	57.74	2.78	61.51	4.15
		30	72.65	3.78	80.57	5.19

Table S2. Shear elastic modulus of the triceps brachii muscle obtained in both series of *experiment* 1. SD, standard deviation over ten measurements; F, female; M, male.

Triceps brachii			1st series		2nd series	
# Subject	Sex (F/M)	% MVC	c_{55} (kPa)	SD	c_{55} (kPa)	SD
1	F	0	6.67	0.46	6.26	0.42
		10	7.49	0.52	7.41	0.96
		20	6.15	0.42	5.79	0.92
		30	6.66	1.05	6.11	0.55
2	M	0	6.34	0.42	6.48	0.43
		10	6.33	0.4	6.46	0.35
		20	6.24	0.36	7.03	0.75
		30	5.96	0.37	6.0	0.47
3	F	0	7.56	0.44	7.39	0.68
		10	9.13	0.68	7.68	0.65
		20	7.22	0.78	5.9	0.31
		30	6.77	1.01	6.12	0.28
4	M	0	9.96	0.68	9.34	0.64
		10	10.38	0.94	9.22	0.48
		20	12.6	0.82	10.73	0.98
		30	13.54	1.58	13.26	2.01
5	M	0	8.41	0.7	8.86	0.63
		10	8.23	0.46	8.72	0.67
		20	5.45	0.65	5.64	0.64
		30	7.8	0.26	7.98	0.28
6	F	0	5.01	0.23	4.81	0.29
		10	5.06	0.18	4.74	0.11
		20	4.9	0.28	4.65	0.3
		30	5.16	0.24	4.96	0.09
7	M	0	10.12	1.29	12.62	1.22
		10	8.7	0.5	10.35	1.12
		20	9.68	1.01	11.46	0.95
		30	13.84	1.44	15.97	1.87
8	M	0	9.36	0.87	7.95	0.86
		10	10.84	0.86	9.79	0.79
		20	14.67	3.05	12.03	0.84
		30	15.06	1.8	13.93	1.84
9	F	0	4.42	0.27	4.46	0.27
		10	3.92	0.34	4.51	0.33
		20	4.65	0.43	4.77	0.66
		30	4.3	0.38	4.6	0.74

10	M	0	7.18	1.06	6.88	0.58
		10	13.39	1.49	10.13	2.05
		20	13.49	2.79	11.01	0.72
		30	13.00	1.51	11.56	1.6
11	F	0	8.22	0.52	8.19	0.64
		10	7.78	0.71	8.31	0.84
		20	7.33	0.31	7.58	0.48
		30	7.66	0.75	7.64	0.73
12	M	0	13.37	0.94	13.72	1.1
		10	11.68	1.26	12.77	1.82
		20	13.00	1.42	15.03	1.25
		30	13.61	1.12	13.65	1.34
13	F	0	9.17	0.77	9.86	0.69
		10	7.2	0.52	8.48	0.49
		20	9.47	0.48	9.96	1.5
		30	9.97	0.78	9.93	1.73
14	F	0	10.19	0.42	10.65	0.95
		10	12.0	0.91	13.42	0.79
		20	10.19	0.49	10.3	0.89
		30	11.31	1.27	11.65	1.25
15	F	0	4.67	0.35	4.61	0.47
		10	4.31	0.76	4.22	0.31
		20	4.96	0.76	4.98	0.46
		30	5.44	0.9	4.15	0.66
16	F	0	9.26	0.36	9.51	0.61
		10	7.36	1.37	8.04	0.75
		20	7.18	0.45	8.49	0.38
		30	7.28	0.46	8.21	0.43
17	M	0	8.54	0.92	8.36	0.97
		10	6.97	0.41	6.3	0.81
		20	7.78	1.26	7.28	1.42
		30	10.04	2.13	8.35	1.08
18	M	0	9.6	0.97	10.86	0.44
		10	12.73	0.97	15.37	1.08
		20	10.66	0.6	12.57	2.97
		30	10.56	0.79	12.58	1.19

Table S3. Absolute values of the EMG RMS recorded in the biceps brachii of each subject during the first series of *experiment* 2. F, female; M, male.

Series #1	EMG RMS (mV)					
			**# Subject**			
**time (s)**	**1 (M)**	**2 (M)**	**3 (F)**	**4 (F)**	**5 (M)**	**6 (M)**
0.69	17.35	21.79	43.48	93.97	20.01	--
1.38	22.84	23.25	46.35	96.49	19.67	14.19
2.08	19.7	22.43	53.87	94.45	19.2	11.66
2.77	37.57	24.63	60.74	91.06	20.55	--
3.46	36.52	23.0	51.96	97.33	33.11	--
4.15	56.65	26.58	67.78	136.79	39.42	19.41
4.85	61.6	27.0	80.72	114.4	38.5	--
5.54	77.47	28.52	76.44	153.5	53.62	22.7
6.23	73.17	30.4	110.49	166.09	70.13	31.02
6.92	82.25	30.77	98.51	130.18	65.14	34.46
7.62	76.61	32.6	108.32	160.14	63.84	31.37
8.31	86.41	56.37	128.03	191.32	67.79	34.91
9.0	87.36	79.12	156.91	228.1	66.92	42.84
9.69	85.33	89.76	221.28	287.24	74.03	44.24
10.38	85.89	105.61	195.09	276.2	63.4	67.67
11.08	78.94	134.32	203.39	356.62	77.17	70.93
11.77	89.02	145.0	254.0	402.48	71.57	92.09
12.46	99.21	150.78	224.75	397.72	82.14	144.11
13.15	100.1	162.13	235.31	558.62	110.46	127.42
13.85	119.54	163.25	243.07	593.96	113.8	137.41
14.54	121.43	189.37	328.85	625.71	135.89	159.94
15.23	137.33	189.45	337.59	757.15	150.2	181.14
15.92	129.05	225.39	409.92	809.71	164.77	224.19
16.62	157.95	218.16	399.43	931.0	190.69	265.77
17.31	151.8	255.4	485.18	740.69	190.65	233.54
18.0	129.41	257.09	414.66	931.14	188.77	240.72
18.69	199.68	299.27	505.04	1003.26	186.08	257.02
19.38	214.54	363.91	523.64	1020.1	220.61	320.52
20.08	228.89	360.63	565.21	1038.14	276.78	338.73
20.77	232.36	426.74	609.52	1073.73	361.09	337.13
21.46	206.15	510.38	743.85	1208.78	517.53	387.69
22.15	251.22	500.24	826.35	1203.03	532.21	438.22
22.85	244.42	464.8	937.47	1277.89	559.36	500.67
23.54	248.15	516.59	804.08	1306.03	473.19	393.64
24.23	276.89	462.2	852.73	1232.42	558.94	479.82
24.92	262.95	486.95	900.9	1104.17	531.89	384.45
25.62	292.01	493.61	847.04	990.6	552.91	389.75
26.31	268.72	451.43	851.33	1109.61	570.79	450.49

27.0	257.97	530.13	748.81	1077.99	497.04	415.42
27.69	233.38	524.37	743.81	1095.07	468.54	--
28.38	232.38	542.76	708.3	1064.61	405.1	436.64
29.08	231.92	503.16	744.68	1097.89	360.32	411.39
29.77	189.48	456.85	775.82	968.33	276.35	360.06
30.46	179.31	485.61	613.75	1062.03	233.21	363.88
31.15	179.38	390.35	710.71	1044.65	226.05	391.47
31.85	158.4	332.42	691.19	1042.13	198.71	409.93
32.54	159.92	333.54	569.22	874.01	173.51	338.15
33.23	151.71	303.43	579.91	816.93	175.02	326.14
33.92	154.61	302.01	538.04	916.13	186.64	274.88
34.62	147.24	277.87	489.79	889.66	157.78	244.28
35.31	155.33	267.65	484.32	828.13	152.29	241.77
36.0	126.01	279.77	465.09	839.06	136.58	168.2
36.69	135.63	206.26	430.81	691.22	125.1	186.41
37.38	120.57	213.1	456.42	502.75	101.37	177.2
38.08	117.65	222.62	376.88	552.23	108.32	173.98
38.77	114.01	211.15	403.31	657.24	97.95	148.69
39.46	100.83	220.21	382.77	493.49	89.93	137.36
40.15	101.73	211.52	334.21	272.53	60.38	126.44
40.85	79.49	160.92	351.51	381.69	62.37	107.45
41.54	71.91	171.36	322.58	210.05	49.69	121.34
42.23	73.75	131.51	348.26	175.73	52.04	127.9
42.92	64.94	99.51	415.46	144.7	55.59	140.41
43.62	61.39	103.98	351.49	109.98	47.2	106.28
44.31	53.27	96.08	273.01	68.17	51.28	107.61
45.0	54.74	77.58	328.99	24.22	37.71	114.42

Table S4. Absolute values of the shear elastic modulus (c_{55}) recorded in the biceps brachii of each subject during the first series of *experiment* 2. F, female; M, male.

Series #1	c_{55} (kPa)					
	**# Subject**					
**time (s)**	**1 (M)**	**2 (M)**	**3 (F)**	**4 (F)**	**5 (M)**	**6 (M)**
0.69	17.14	22.81	10.71	3.89	16.1	--
1.38	17.22	25.88	12.15	3.6	15.35	6.35
2.08	17.7	25.59	9.84	3.85	16.64	6.11
2.77	19.26	27.21	12.09	3.64	16.81	--
3.46	19.71	29.27	13.23	2.31	20.82	--
4.15	24.55	33.07	11.77	2.07	30.68	8.91
4.85	24.68	36.02	11.15	2.68	34.89	--
5.54	26.6	37.23	15.61	3.01	45.42	11.36
6.23	26.56	38.88	17.63	3.13	56.13	14.67
6.92	26.89	36.7	15.14	14.47	65.95	16.34
7.62	29.17	36.26	20.26	13.38	60.49	16.59
8.31	29.21	34.29	29.69	14.58	64.26	20.79
9.0	30.55	35.87	35.51	13.22	71.95	24.73
9.69	24.91	36.84	37.13	15.63	75.92	25.15
10.38	27.84	35.79	46.41	15.8	67.72	26.78
11.08	30.37	34.77	55.71	18.95	71.3	33.69
11.77	27.36	34.58	68.07	19.22	62.05	31.2
12.46	36.6	38.7	74.68	30.45	67.18	31.52
13.15	40.08	44.08	79.39	23.21	68.85	32.68
13.85	43.31	52.94	84.03	29.65	69.64	39.54
14.54	54.69	58.74	80.51	48.89	68.6	39.74
15.23	58.67	64.24	83.76	36.56	93.62	47.01
15.92	61.22	67.31	94.69	47.96	100.98	51.59
16.62	60.28	69.25	96.83	63.94	109.76	51.25
17.31	64.41	74.45	99.31	74.91	111.29	50.54
18.0	67.23	78.9	114.92	89.78	119.81	54.8
18.69	70.83	83.25	115.59	94.13	125.13	63.41
19.38	68.67	89.57	167.06	89.73	134.16	74.32
20.08	65.72	89.02	172.75	84.06	155.08	68.99
20.77	69.79	90.12	181.05	94.17	170.00	78.5
21.46	70.34	90.91	173.08	97.54	187.04	79.28
22.15	68.85	89.86	181.52	97.15	173.00	70.67
22.85	76.55	95.79	185.73	102.68	171.4	68.46
23.54	75.35	93.88	184.08	99.76	168.28	72.35
24.23	77.16	90.05	155.71	103.01	160.98	73.3
24.92	80.09	93.02	181.24	90.09	168.19	75.12
25.62	76.26	90.42	170.02	95.66	154.33	84.05

26.31	76.44	86.52	172.66	83.49	138.88	79.29
27.0	76.06	84.78	166.8	80.58	126.36	87.71
27.69	67.43	83.11	205.23	86.09	123.46	--
28.38	69.96	94.03	200.35	80.7	111.39	80.66
29.08	69.17	90.28	184.89	73.36	110.64	62.05
29.77	68.85	90.43	184.09	73.15	120.79	60.45
30.46	67.34	87.7	185.85	70.03	119.85	73.87
31.15	64.81	95.17	184.31	73.76	113.59	51.32
31.85	57.75	81.43	175.52	65.83	96.31	54.28
32.54	56.86	71.63	163.5	67.32	82.04	57.19
33.23	50.79	65.66	154.17	68.97	77.56	44.4
33.92	49.58	72.29	174.44	60.86	73.61	38.23
34.62	45.21	61.32	157.85	59.27	67.3	34.38
35.31	41.77	58.26	138.36	56.81	64.12	33.07
36.0	37.29	55.29	109.61	47.46	66.91	30.14
36.69	35.56	42.59	110.41	46.41	54.59	24.22
37.38	31.55	44.96	98.07	42.58	55.36	27.3
38.08	34.75	39.72	96.77	41.83	50.52	28.17
38.77	28.13	38.08	113.71	36.9	43.8	26.35
39.46	29.36	35.62	114.94	35.04	43.13	22.51
40.15	23.01	30.44	81.01	35.6	42.75	22.67
40.85	24.86	29.43	73.97	21.04	39.95	17.91
41.54	22.33	25.3	69.39	21.45	36.65	15.38
42.23	17.22	24.79	74.73	22.62	28.63	15.98
42.92	12.19	20.34	76.98	24.25	23.84	14.86
43.62	11.09	18.94	75.16	18.86	21.19	16.05
44.31	10.36	19.04	64.34	19.24	18.94	12.76
45.0	10.2	17.4	67.41	19.27	15.27	14.58

Table S5. Absolute values of the EMG RMS recorded in the biceps brachii of each subject during the second series of *experiment* 2. F, female; M, male.

Series #2	EMG RMS (mV)					
	**# Subject**					
**time (s)**	**1 (M)**	**2 (M)**	**3 (F)**	**4 (F)**	**5 (M)**	**6 (M)**
0.69	--	24.17	--	84.29	48.30	51.17
1.38	33.03	27.12	98.72	118.26	40.52	52.97
2.08	42.74	26.81	100.16	111.63	34.35	46.68
2.77	46.77	28.80	104.10	126.92	39.93	59.05
3.46	57.56	32.11	102.73	126.25	39.46	51.75
4.15	61.86	45.08	106.46	134.05	50.39	70.24
4.85	76.81	68.86	123.97	135.23	59.13	88.74
5.54	77.10	89.73	118.25	163.91	70.70	74.59
6.23	73.37	86.00	135.25	185.18	67.91	85.80
6.92	78.52	109.27	122.78	223.94	76.27	99.39
7.62	80.91	118.19	135.64	282.12	75.58	109.46
8.31	96.16	134.41	186.90	308.03	84.37	131.98
9.0	97.75	151.21	232.09	336.86	109.20	139.62
9.69	80.26	140.90	211.99	412.37	136.11	135.94
10.38	95.98	157.15	243.34	361.52	115.12	158.09
11.08	89.48	148.78	256.96	363.53	128.78	199.85
11.77	125.87	182.95	309.06	512.20	133.16	204.94
12.46	153.62	178.57	262.05	548.19	171.06	215.62
13.15	130.43	238.09	309.80	543.14	189.58	260.96
13.85	128.01	238.14	388.53	660.18	195.78	295.50
14.54	165.38	241.09	354.68	794.67	197.60	327.48
15.23	202.02	269.15	442.14	770.12	205.88	392.52
15.92	201.71	282.13	487.39	867.30	172.50	388.95
16.62	222.81	351.23	476.47	772.49	216.68	--
17.31	158.66	351.56	664.61	881.77	--	--
18.0	196.77	362.16	757.40	952.26	--	357.14
18.69	199.70	378.88	729.87	1053.12	--	416.98
19.38	199.11	420.14	725.89	1058.25	--	520.70
20.08	214.22	469.83	--	1180.71	--	475.15
20.77	323.78	475.06	968.26	1163.09	468.90	--
21.46	307.70	479.72	--	1181.06	538.92	533.18
22.15	300.64	512.66	--	1256.15	570.51	--
22.85	314.68	502.20	--	1286.37	531.86	492.56
23.54	252.21	525.51	--	1392.59	549.94	469.39
24.23	299.72	--	--	--	522.20	452.28
24.92	289.93	508.68	--	--	545.34	463.06
25.62	284.58	483.36	--	1462.17	315.99	550.48
26.31	281.79	527.76	1123.35	--	160.68	529.58

27.0	277.53	493.62	--	1431.02	203.08	472.93
27.69	249.82	429.63	1068.59	1514.97	191.72	452.95
28.38	275.17	452.07	890.97	1442.71	210.10	471.88
29.08	245.92	477.20	850.39	1494.46	204.54	342.72
29.77	243.90	471.44	836.70	1437.74	200.51	324.68
30.46	249.26	441.96	923.25	1483.70	182.17	315.69
31.15	216.98	437.79	833.46	1459.24	196.38	300.91
31.85	185.69	343.34	824.11	1438.26	178.09	238.86
32.54	182.93	377.77	718.81	1496.57	180.21	254.54
33.23	174.64	331.28	800.99	1360.61	163.04	245.79
33.92	164.61	293.72	725.43	1454.31	139.80	181.83
34.62	199.67	261.82	709.90	1371.63	139.57	160.76
35.31	174.38	228.62	621.52	1234.48	104.15	168.51
36.0	154.36	226.94	547.17	1247.42	100.47	149.04
36.69	146.87	207.23	524.53	1213.34	97.52	131.59
37.38	127.64	196.05	509.67	1091.22	85.84	155.68
38.08	135.00	191.16	510.88	1253.45	60.21	147.52
38.77	100.02	174.56	441.12	1183.58	61.23	127.78
39.46	86.49	155.61	412.06	1197.10	41.20	107.88
40.15	72.12	190.95	454.91	1181.78	31.23	101.04
40.85	80.89	183.09	442.76	1112.55	39.29	86.40
41.54	74.57	151.36	384.25	1172.64	22.03	82.59
42.23	70.84	114.64	389.16	1049.71	18.93	68.28
42.92	60.44	108.10	424.27	1066.01	12.64	74.46
43.62	66.59	95.73	353.91	985.45	11.47	74.82
44.31	38.29	103.47	364.44	1008.63	12.13	69.11
45.0	3.20	91.39	303.91	984.56	13.63	78.82

Table S6. Absolute values of the shear elastic modulus (c_{55}) recorded in the biceps brachii of each subject during the second series of *experiment* 2. F, female; M, male.

Series #2	c_{55} (kPa)					
			**# Subject**			
**time (s)**	**1 (M)**	**2 (M)**	**3 (F)**	**4 (F)**	**5 (M)**	**6 (M)**
0.69	--	15.81	--	2.98	16.01	18.66
1.38	12.34	15.3	4.83	2.22	17.04	17.49
2.08	11.85	15.36	5.02	3.04	16.98	16.9
2.77	11.16	18.21	5.98	3.09	24.93	18.73
3.46	11.34	22.72	4.8	2.99	29.7	19.96
4.15	15.46	29.05	6.00	2.85	34.15	19.63
4.85	20.87	29.99	5.84	2.77	48.67	22.04
5.54	24.35	31.25	7.5	2.66	75.35	20.58
6.23	37.67	33.83	8.6	3.46	85.41	22.57
6.92	48.57	35.23	13.53	3.98	78.79	21.28
7.62	45.8	33.00	14.27	4.17	63.67	22.92
8.31	50.76	32.69	14.13	4.74	66.1	19.37
9.0	49.54	30.22	15.14	8.82	65.89	17.97
9.69	51.79	29.18	21.04	13.81	71.14	20.93
10.38	57.42	28.84	22.05	14.31	66.03	24.44
11.08	60.61	27.71	24.21	15.00	68.06	27.17
11.77	58.62	30.29	28.09	18.13	66.83	20.8
12.46	61.38	31.15	30.29	21.34	71.34	29.43
13.15	56.4	32.89	36.47	21.46	76.32	21.51
13.85	61.97	36.6	48.58	24.24	107.2	28.26
14.54	65.62	45.81	59.47	67.5	112.29	27.31
15.23	66.91	48.81	85.77	48.93	99.94	36.77
15.92	65.31	53.69	108.38	59.32	115.2	47.55
16.62	70.55	57.18	152.86	66.2	115.23	--
17.31	74.42	63.31	139.52	100.07	--	--
18.0	66.8	69.8	171.14	80.83	--	53.11
18.69	67.18	78.34	185.04	84.82	--	60.34
19.38	76.26	79.85	187.96	95.58	--	63.51
20.08	74.82	78.15	--	100.16	--	70.82
20.77	70.98	81.92	192.21	101.3	134.63	--
21.46	76.83	84.85	--	95.9	138.94	76.77
22.15	74.03	92.00	--	98.41	142.83	--
22.85	74.58	85.98	--	98.16	152.76	75.93
23.54	81.18	84.29	--	114.36	146.49	72.51
24.23	85.41	--	--	--	148.13	67.31
24.92	75.17	86.99	--	--	159.79	65.99
25.62	83.2	87.24	--	116.3	158.84	70.87

26.31	77.37	88.68	165.23	--	152.39	67.99
27.0	77.36	88.43	--	105.91	147.69	62.96
27.69	74.41	85.02	182.73	96.66	135.54	68.76
28.38	73.89	77.1	178.03	93.09	116.69	66.7
29.08	68.41	80.09	167.61	101.44	108.54	62.53
29.77	64.63	79.47	172.76	91.86	115.32	62.48
30.46	59.3	76.69	162.47	90.49	84.71	61.22
31.15	53.81	73.44	151.18	90.89	90.85	60.00
31.85	56.29	70.32	168.48	80.12	91.01	59.06
32.54	53.28	62.93	148.84	73.76	75.26	56.13
33.23	49.44	62.17	165.11	66.66	67.56	47.22
33.92	47.55	59.26	146.46	58.71	68.08	46.42
34.62	47.03	58.87	134.05	56.13	66.41	41.21
35.31	44.9	49.45	140.84	53.95	61.25	43.55
36.0	39.65	42.81	143.66	54.89	54.88	43.5
36.69	37.52	41.37	151.99	50.41	45.74	38.82
37.38	35.13	34.37	111.71	53.97	45.33	40.18
38.08	32.64	29.95	97.94	58.25	48.35	45.96
38.77	26.29	27.27	91.13	56.53	39.48	36.84
39.46	20.22	24.44	84.1	57.23	40.88	40.85
40.15	15.76	25.88	57.41	53.26	35.86	42.72
40.85	19.46	22.72	56.84	51.12	34.68	44.65
41.54	21.6	20.4	50.49	41.02	27.62	37.35
42.23	15.71	18.8	54.77	35.98	30.89	27.7
42.92	15.85	18.02	43.74	27.78	41.22	41.15
43.62	6.8	14.6	38.47	24.4	18.08	25.8
44.31	2.23	12.89	25.48	20.54	17.2	25.5
45.0	2.46	11.82	39.6	17.39	19.72	17.68

Table S7. Normalized values of the EMG RMS recorded in the biceps brachii of each subject during the first series of *experiment* 2. The values were normalized with respect to the maximal RMS along the ramp. F, female; M, male.

Series #1	Normalized EMG RMS					
	**# Subject**					
**time (s)**	**1 (M)**	**2 (M)**	**3 (F)**	**4 (F)**	**5 (M)**	**6 (M)**
0.69	0.06	0.04	0.05	0.07	0.04	--
1.38	0.08	0.04	0.05	0.07	0.03	0.03
2.08	0.07	0.04	0.06	0.07	0.03	0.02
2.77	0.13	0.05	0.06	0.07	0.04	--
3.46	0.13	0.04	0.06	0.07	0.06	--
4.15	0.19	0.05	0.07	0.10	0.07	0.04
4.85	0.21	0.05	0.09	0.09	0.07	--
5.54	0.27	0.05	0.08	0.12	0.09	0.05
6.23	0.25	0.06	0.12	0.13	0.12	0.06
6.92	0.28	0.06	0.11	0.10	0.11	0.07
7.62	0.26	0.06	0.12	0.12	0.11	0.06
8.31	0.30	0.10	0.14	0.15	0.12	0.07
9.0	0.30	0.15	0.17	0.17	0.12	0.09
9.69	0.29	0.17	0.24	0.22	0.13	0.09
10.38	0.29	0.19	0.21	0.21	0.11	0.14
11.08	0.27	0.25	0.22	0.27	0.14	0.14
11.77	0.30	0.27	0.27	0.31	0.13	0.18
12.46	0.34	0.28	0.24	0.30	0.14	0.29
13.15	0.34	0.30	0.25	0.43	0.19	0.25
13.85	0.41	0.30	0.26	0.45	0.20	0.27
14.54	0.42	0.35	0.35	0.48	0.24	0.32
15.23	0.47	0.35	0.36	0.58	0.26	0.36
15.92	0.44	0.42	0.44	0.62	0.29	0.45
16.62	0.54	0.40	0.43	0.71	0.33	0.53
17.31	0.52	0.47	0.52	0.57	0.33	0.47
18.0	0.44	0.47	0.44	0.71	0.33	0.48
18.69	0.68	0.55	0.54	0.77	0.33	0.51
19.38	0.73	0.67	0.56	0.78	0.39	0.64
20.08	0.78	0.66	0.60	0.79	0.48	0.68
20.77	0.80	0.79	0.65	0.82	0.63	0.67
21.46	0.71	0.94	0.79	0.93	0.91	0.77
22.15	0.86	0.92	0.88	0.92	0.93	0.88
22.85	0.84	0.86	1.00	0.98	0.98	1.00
23.54	0.85	0.95	0.86	1.00	0.83	0.79
24.23	0.95	0.85	0.91	0.94	0.98	0.96
24.92	0.90	0.90	0.96	0.85	0.93	0.77

25.62	1.00	0.91	0.90	0.76	0.97	0.78
26.31	0.92	0.83	0.91	0.85	1.00	0.90
27.0	0.88	0.98	0.80	0.83	0.87	0.83
27.69	0.80	0.97	0.79	0.84	0.82	--
28.38	0.80	1.00	0.76	0.82	0.71	0.87
29.08	0.79	0.93	0.79	0.84	0.63	0.82
29.77	0.65	0.84	0.83	0.74	0.48	0.72
30.46	0.61	0.89	0.65	0.81	0.41	0.73
31.15	0.61	0.72	0.76	0.80	0.40	0.78
31.85	0.54	0.61	0.74	0.80	0.35	0.82
32.54	0.55	0.61	0.61	0.67	0.30	0.68
33.23	0.52	0.56	0.62	0.63	0.31	0.65
33.92	0.53	0.56	0.57	0.70	0.33	0.55
34.62	0.50	0.51	0.52	0.68	0.28	0.49
35.31	0.53	0.49	0.52	0.63	0.27	0.48
36.0	0.43	0.52	0.50	0.64	0.24	0.34
36.69	0.46	0.38	0.46	0.53	0.22	0.37
37.38	0.41	0.39	0.49	0.38	0.18	0.35
38.08	0.40	0.41	0.40	0.42	0.19	0.35
38.77	0.39	0.39	0.43	0.50	0.17	0.30
39.46	0.35	0.41	0.41	0.38	0.16	0.27
40.15	0.35	0.39	0.36	0.21	0.11	0.25
40.85	0.27	0.30	0.37	0.29	0.11	0.21
41.54	0.25	0.32	0.34	0.16	0.09	0.24
42.23	0.25	0.24	0.37	0.13	0.09	0.26
42.92	0.22	0.18	0.44	0.11	0.10	0.28
43.62	0.21	0.19	0.37	0.08	0.08	0.21
44.31	0.18	0.18	0.29	0.05	0.09	0.21
45.0	0.19	0.14	0.35	0.02	0.07	0.23

Table S8. Normalized values of the shear elastic modulus (c_{55}) recorded in the biceps brachii of each subject during the first series of *experiment* 2. The values were normalized with respect to the maximal c_{55} along the ramp. F, female; M, male.

Series #1	Normalized c_{55}					
			**# Subject**			
**time (s)**	**1 (M)**	**2 (M)**	**3 (F)**	**4 (F)**	**5 (M)**	**6 (M)**
0.69	0.21	0.24	0.05	0.04	0.09	--
1.38	0.22	0.27	0.06	0.03	0.08	0.07
2.08	0.22	0.27	0.05	0.04	0.09	0.07
2.77	0.24	0.28	0.06	0.04	0.09	--
3.46	0.25	0.31	0.06	0.02	0.11	--
4.15	0.31	0.35	0.06	0.02	0.16	0.10
4.85	0.31	0.38	0.05	0.03	0.19	--
5.54	0.33	0.39	0.08	0.03	0.24	0.13
6.23	0.33	0.41	0.09	0.03	0.30	0.17
6.92	0.34	0.38	0.07	0.14	0.35	0.19
7.62	0.36	0.38	0.10	0.13	0.32	0.19
8.31	0.36	0.36	0.14	0.14	0.34	0.24
9.0	0.38	0.37	0.17	0.13	0.38	0.28
9.69	0.31	0.38	0.18	0.15	0.41	0.29
10.38	0.35	0.37	0.23	0.15	0.36	0.31
11.08	0.38	0.36	0.27	0.18	0.38	0.38
11.77	0.34	0.36	0.33	0.19	0.33	0.36
12.46	0.46	0.40	0.36	0.30	0.36	0.36
13.15	0.50	0.46	0.39	0.23	0.37	0.37
13.85	0.54	0.55	0.41	0.29	0.37	0.45
14.54	0.68	0.61	0.39	0.47	0.37	0.45
15.23	0.73	0.67	0.41	0.35	0.50	0.54
15.92	0.76	0.70	0.46	0.47	0.54	0.59
16.62	0.75	0.72	0.47	0.62	0.59	0.58
17.31	0.80	0.78	0.48	0.73	0.60	0.58
18.0	0.84	0.82	0.56	0.87	0.64	0.62
18.69	0.88	0.87	0.56	0.91	0.67	0.72
19.38	0.86	0.94	0.81	0.87	0.72	0.85
20.08	0.82	0.93	0.84	0.82	0.83	0.79
20.77	0.87	0.94	0.88	0.91	0.91	0.89
21.46	0.88	0.95	0.84	0.95	1.00	0.90
22.15	0.86	0.94	0.88	0.94	0.92	0.81
22.85	0.96	1.00	0.90	1.00	0.92	0.78
23.54	0.94	0.98	0.90	0.97	0.90	0.82
24.23	0.96	0.94	0.76	1.00	0.86	0.84
24.92	1.00	0.97	0.88	0.87	0.90	0.86

25.62	0.95	0.94	0.83	0.93	0.83	0.96
26.31	0.95	0.90	0.84	0.81	0.74	0.90
27.0	0.95	0.89	0.81	0.78	0.68	1.00
27.69	0.84	0.87	1.00	0.84	0.66	--
28.38	0.87	0.98	0.98	0.78	0.60	0.92
29.08	0.86	0.94	0.90	0.71	0.59	0.71
29.77	0.86	0.94	0.90	0.71	0.65	0.69
30.46	0.84	0.92	0.91	0.68	0.64	0.84
31.15	0.81	0.99	0.90	0.72	0.61	0.59
31.85	0.72	0.85	0.86	0.64	0.51	0.62
32.54	0.71	0.75	0.80	0.65	0.44	0.65
33.23	0.63	0.69	0.75	0.67	0.41	0.51
33.92	0.62	0.75	0.85	0.59	0.39	0.44
34.62	0.56	0.64	0.77	0.58	0.36	0.39
35.31	0.52	0.61	0.67	0.55	0.34	0.38
36.0	0.47	0.58	0.53	0.46	0.36	0.34
36.69	0.44	0.44	0.54	0.45	0.29	0.28
37.38	0.39	0.47	0.48	0.41	0.30	0.31
38.08	0.43	0.41	0.47	0.41	0.27	0.32
38.77	0.35	0.40	0.55	0.36	0.23	0.30
39.46	0.37	0.37	0.56	0.34	0.23	0.26
40.15	0.29	0.32	0.39	0.35	0.23	0.26
40.85	0.31	0.31	0.36	0.20	0.21	0.20
41.54	0.28	0.26	0.34	0.21	0.20	0.18
42.23	0.22	0.26	0.36	0.22	0.15	0.18
42.92	0.15	0.21	0.38	0.24	0.13	0.17
43.62	0.14	0.20	0.37	0.18	0.11	0.18
44.31	0.13	0.20	0.31	0.19	0.10	0.15
45.0	0.13	0.18	0.33	0.19	0.08	0.17

Table 9. Normalized values of the EMG RMS recorded in the biceps brachii of each subject during the second series of *experiment* 2. The values were normalized with respect to the maximal RMS along the ramp. F, female; M, male.

Serie #2	Normalized EMG RMS					
			**# Subject**			
**time (s)**	**1 (M)**	**2 (M)**	**3 (F)**	**4 (F)**	**5 (M)**	**6 (M)**
0.69	--	0.05	--	0.06	0.08	0.09
1.38	0.10	0.05	0.09	0.08	0.07	0.10
2.08	0.13	0.05	0.09	0.07	0.06	0.08
2.77	0.14	0.05	0.09	0.08	0.07	0.11
3.46	0.18	0.06	0.09	0.08	0.07	0.09
4.15	0.19	0.09	0.09	0.09	0.09	0.13
4.85	0.24	0.13	0.11	0.09	0.10	0.16
5.54	0.24	0.17	0.11	0.11	0.12	0.14
6.23	0.23	0.16	0.12	0.12	0.12	0.16
6.92	0.24	0.21	0.11	0.15	0.13	0.18
7.62	0.25	0.22	0.12	0.19	0.13	0.20
8.31	0.30	0.25	0.17	0.20	0.15	0.24
9.0	0.30	0.29	0.21	0.22	0.19	0.25
9.69	0.25	0.27	0.19	0.27	0.24	0.25
10.38	0.30	0.30	0.22	0.24	0.20	0.29
11.08	0.28	0.28	0.23	0.24	0.23	0.36
11.77	0.39	0.35	0.28	0.34	0.23	0.37
12.46	0.47	0.34	0.23	0.36	0.30	0.39
13.15	0.40	0.45	0.28	0.36	0.33	0.47
13.85	0.40	0.45	0.35	0.44	0.34	0.54
14.54	0.51	0.46	0.32	0.52	0.35	0.59
15.23	0.62	0.51	0.39	0.51	0.36	0.71
15.92	0.62	0.53	0.43	0.57	0.30	0.71
16.62	0.69	0.67	0.42	0.51	0.38	--
17.31	0.49	0.67	0.59	0.58	--	--
18.0	0.61	0.69	0.67	0.63	--	0.65
18.69	0.62	0.72	0.65	0.70	--	0.76
19.38	0.61	0.80	0.65	0.70	--	0.95
20.08	0.66	0.89	--	0.78	--	0.86
20.77	1.00	0.90	0.86	0.77	0.82	--
21.46	0.95	0.91	--	0.78	0.94	0.97
22.15	0.93	0.97	--	0.83	1.00	--
22.85	0.97	0.95	--	0.85	0.93	0.89
23.54	0.78	1.00	--	0.92	0.96	0.85
24.23	0.93	--	--	--	0.92	0.82
24.92	0.90	0.96	--	--	0.96	0.84

25.62	0.88	0.92	--	0.97	0.55	1.00
26.31	0.87	1.00	1.00	--	0.28	0.96
27.0	0.86	0.94	--	0.94	0.36	0.86
27.69	0.77	0.81	0.95	1.00	0.34	0.82
28.38	0.85	0.86	0.79	0.95	0.37	0.86
29.08	0.76	0.90	0.76	0.99	0.36	0.62
29.77	0.75	0.89	0.74	0.95	0.35	0.59
30.46	0.77	0.84	0.82	0.98	0.32	0.57
31.15	0.67	0.83	0.74	0.96	0.34	0.55
31.85	0.57	0.65	0.73	0.95	0.31	0.43
32.54	0.56	0.72	0.64	0.99	0.32	0.46
33.23	0.54	0.63	0.71	0.90	0.29	0.45
33.92	0.51	0.56	0.65	0.96	0.25	0.33
34.62	0.62	0.50	0.63	0.91	0.24	0.29
35.31	0.54	0.43	0.55	0.81	0.18	0.31
36.0	0.48	0.43	0.49	0.82	0.18	0.27
36.69	0.45	0.39	0.47	0.80	0.17	0.24
37.38	0.39	0.37	0.45	0.72	0.15	0.28
38.08	0.42	0.36	0.45	0.83	0.11	0.27
38.77	0.31	0.33	0.39	0.78	0.11	0.23
39.46	0.27	0.29	0.37	0.79	0.07	0.20
40.15	0.22	0.36	0.40	0.78	0.05	0.18
40.85	0.25	0.35	0.39	0.73	0.07	0.16
41.54	0.23	0.29	0.34	0.77	0.04	0.15
42.23	0.22	0.22	0.35	0.69	0.03	0.12
42.92	0.19	0.20	0.38	0.70	0.02	0.14
43.62	0.21	0.18	0.32	0.65	0.02	0.14
44.31	0.12	0.20	0.32	0.67	0.02	0.13
45.0	0.01	0.17	0.27	0.65	0.02	0.14

Table S10. Normalized values of the shear elastic modulus (c_{55}) recorded in the biceps brachii of each subject during the second series of *experiment* 2. The values were normalized with respect to the maximal c_{55} along the ramp. F, female; M, male.

Serie #2	c_{55} normalizado					
			**# Subject**			
**time (s)**	**1 (M)**	**2 (M)**	**3 (F)**	**4 (F)**	**5 (M)**	**6 (M)**
0.69	--	0.17	--	0.03	0.10	0.24
1.38	0.14	0.17	0.03	0.02	0.11	0.23
2.08	0.14	0.17	0.03	0.03	0.11	0.22
2.77	0.13	0.20	0.03	0.03	0.16	0.24
3.46	0.13	0.25	0.02	0.03	0.19	0.26
4.15	0.18	0.32	0.03	0.02	0.21	0.26
4.85	0.24	0.33	0.03	0.02	0.30	0.29
5.54	0.29	0.34	0.04	0.02	0.47	0.27
6.23	0.44	0.37	0.04	0.03	0.53	0.29
6.92	0.57	0.38	0.07	0.03	0.49	0.28
7.62	0.54	0.36	0.07	0.04	0.40	0.30
8.31	0.59	0.36	0.07	0.04	0.41	0.25
9.0	0.58	0.33	0.08	0.08	0.41	0.23
9.69	0.61	0.32	0.11	0.12	0.45	0.27
10.38	0.67	0.31	0.11	0.12	0.41	0.32
11.08	0.71	0.30	0.13	0.13	0.43	0.35
11.77	0.69	0.33	0.15	0.16	0.42	0.27
12.46	0.72	0.34	0.16	0.18	0.45	0.38
13.15	0.66	0.36	0.19	0.18	0.48	0.28
13.85	0.73	0.40	0.25	0.21	0.67	0.37
14.54	0.77	0.50	0.31	0.58	0.70	0.36
15.23	0.78	0.53	0.45	0.42	0.63	0.48
15.92	0.76	0.58	0.56	0.51	0.72	0.62
16.62	0.83	0.62	0.80	0.57	0.72	--
17.31	0.87	0.69	0.73	0.86	--	--
18.0	0.78	0.76	0.89	0.70	--	0.69
18.69	0.79	0.85	0.96	0.73	--	0.79
19.38	0.89	0.87	0.98	0.82	--	0.83
20.08	0.88	0.85	--	0.86	--	0.92
20.77	0.83	0.89	1.00	0.87	0.84	--
21.46	0.90	0.92	--	0.82	0.87	1.00
22.15	0.87	1.00	--	0.85	0.89	--
22.85	0.87	0.93	--	0.84	0.96	0.99
23.54	0.95	0.92	--	0.98	0.92	0.94
24.23	1.00	--	--	--	0.93	0.88

24.92	0.88	0.95	--	--	1.00	0.86
25.62	0.97	0.95	--	1.00	0.99	0.92
26.31	0.91	0.96	0.86	--	0.95	0.89
27.0	0.91	0.96	--	0.91	0.92	0.82
27.69	0.87	0.92	0.95	0.83	0.85	0.90
28.38	0.87	0.84	0.93	0.80	0.73	0.87
29.08	0.80	0.87	0.87	0.87	0.68	0.81
29.77	0.76	0.86	0.90	0.79	0.72	0.81
30.46	0.69	0.83	0.85	0.78	0.53	0.80
31.15	0.63	0.80	0.79	0.78	0.57	0.78
31.85	0.66	0.76	0.88	0.69	0.57	0.77
32.54	0.62	0.68	0.77	0.63	0.47	0.73
33.23	0.58	0.68	0.86	0.57	0.42	0.62
33.92	0.56	0.64	0.76	0.50	0.43	0.60
34.62	0.55	0.64	0.70	0.48	0.42	0.54
35.31	0.53	0.54	0.73	0.46	0.38	0.57
36.0	0.46	0.47	0.75	0.47	0.34	0.57
36.69	0.44	0.45	0.79	0.43	0.29	0.51
37.38	0.41	0.37	0.58	0.46	0.28	0.52
38.08	0.38	0.33	0.51	0.50	0.30	0.60
38.77	0.31	0.30	0.47	0.49	0.25	0.48
39.46	0.24	0.27	0.44	0.49	0.26	0.53
40.15	0.18	0.28	0.30	0.46	0.22	0.56
40.85	0.23	0.25	0.30	0.44	0.22	0.58
41.54	0.25	0.22	0.26	0.35	0.17	0.49
42.23	0.18	0.20	0.28	0.31	0.19	0.36
42.92	0.19	0.20	0.23	0.24	0.26	0.54
43.62	0.08	0.16	0.20	0.21	0.11	0.34
44.31	0.03	0.14	0.13	0.18	0.11	0.33
45.0	0.03	0.13	0.21	0.15	0.12	0.23
